# The estimated health impact of sodium reduction through food reformulation in Australia: A modeling study

**DOI:** 10.1371/journal.pmed.1003806

**Published:** 2021-10-26

**Authors:** Kathy Trieu, Daisy H. Coyle, Ashkan Afshin, Bruce Neal, Matti Marklund, Jason H. Y. Wu

**Affiliations:** 1 The George Institute for Global Health, Faculty of Medicine, University of New South Wales, Sydney, Australia; 2 University of Washington School of Medicine, Seattle, Washington, United States of America; 3 Department of Epidemiology and Biostatistics, Imperial College London, United Kingdom; 4 Department of Epidemiology, Johns Hopkins Bloomberg School of Public Health, Baltimore, Maryland, United States of America; University of Cambridge, UNITED KINGDOM

## Abstract

**Background:**

The Australian Government recently established sodium targets for packaged foods to encourage voluntary reformulation to reduce population sodium consumption and related diseases. We modeled the health impact of Australia’s sodium reformulation targets and additional likely health gains if more ambitious, yet feasible sodium targets had been adopted instead.

**Methods and findings:**

Using comparative risk assessment models, we estimated the averted deaths, incidence, and disability-adjusted life years (DALYs) from cardiovascular disease (CVD), chronic kidney disease (CKD) and stomach cancer after implementation of (a) Australia’s sodium targets (overall and by individual companies); (b) United Kingdom’s targets (that covers more product categories); and (c) an optimistic scenario (sales-weighted 25th percentile sodium content for each food category included in the UK program). We used nationally representative data to estimate pre- and post-intervention sodium intake, and other key data sources from the Global Burden of Disease study. Full compliance with the Australian government’s sodium targets could prevent approximately 510 deaths/year (95% UI, 335 to 757), corresponding to about 1% of CVD, CKD, and stomach cancer deaths, and prevent some 1,920 (1,274 to 2,600) new cases and 7,240 (5,138 to 10,008) DALYs/year attributable to these diseases. Over half (59%) of deaths prevented is attributed to reformulation by 5 market-dominant companies. Compliance with the UK and optimistic scenario could avert approximately an additional 660 (207 to 1,227) and 1,070 (511 to 1,856) deaths/year, respectively, compared to Australia’s targets. The main limitation of this study (like other modeling studies) is that it does not prove that sodium reformulation programs will prevent deaths and disease events; rather, it provides the best quantitative estimates and the corresponding uncertainty of the potential effect of the different programs to guide the design of policies.

**Conclusions:**

There is significant potential to strengthen Australia’s sodium reformulation targets to improve its health impact. Promoting compliance by market-dominant food companies will be critical to achieving the potential health gains.

## Abstract 2

Kathy Trieu and colleagues model the estimated health impact of sodium reformulation targets in Australia.

## Author summary

**Why was this study done?:**

- High sodium intake increases blood pressure and the risk of heart disease, chronic kidney disease (CKD), and stomach cancer.
- Almost all adults in Australia and worldwide exceed sodium intake recommendations.
- To help reduce population sodium intakes, the Australian Government recently established sodium targets for packaged foods to encourage voluntary reformulation. However, the potential health gains of these targets are not known.

**What did the researchers do and find?:**

- We modeled the potential health gains that could be achieved if all food companies met Australia’s sodium targets and what additional health gains could be achieved if Australia adopted more ambitious targets.
- Using mathematical models, reductions in population sodium intake after implementation of sodium targets were calculated and translated into expected changes in blood pressure and health gains related to heart disease, CKD, and stomach cancer.
- Full compliance with Australia’s sodium targets could prevent approximately 510 deaths/year. Over half of these deaths could be prevented by just 5 food companies complying with the targets. If Australia adopted the sodium targets already implemented in the United Kingdom or a more ambitious, yet feasible set of targets, an additional 660 and 1,070 deaths/year, respectively, could be averted.

**What do these findings mean?:**

- This study demonstrates that national sodium reformulation targets could be an effective strategy to prevent death and disability associated with heart disease, CKD, and stomach cancer.
- Australia’s sodium reformulation targets could achieve greater public health benefits if these targets were mandatory, targeted more food categories, and included more stringent targets.
- To maximize the potential of Australia’s current sodium targets, promoting full compliance across dominant food companies could help to achieve health gains.

## Introduction

Excess sodium consumption causes high blood pressure, a major risk factor for cardiovascular diseases (CVDs) and chronic kidney disease (CKD) [1]. High sodium intake is also related to elevated risk of stomach cancer [2]. Almost all adult populations worldwide consume more sodium than recommended [3]. It was estimated that 3 million noncommunicable disease (NCD) deaths were attributable to high sodium intake in 2017, making it the lead dietary risk factor for deaths worldwide [3]. The World Health Organization (WHO) has therefore urged Member States to reduce mean population sodium intake by 30% by 2025 as part of 9 goals to tackle NCDs [4].

In 2017, WHO recommended sodium reduction through reformulation of food products to contain lower sodium content and setting sodium target levels as a “best buy” intervention—meaning that it was one of the most cost-effective and feasible interventions for the prevention of NCDs [5]. In 2019, 96 countries worldwide had national salt reduction strategies, with the majority consisting of interventions in public settings and food reformulation programs [6,7]. Sodium reduction interventions that engage food companies to reformulate (lower the sodium content in their products) have been the focus in middle- and high-income countries where processed packaged food products are the main source of sodium in the diet, contributing to 70% to 90% of total sodium intakes [6–8]. Over 80% (57 of 68) of reformulation programs involve the establishment of sodium content targets or benchmarks for different food categories, which food companies should work towards [7]. Most national sodium reformulation programs are currently voluntary, with compliance to the suggested sodium targets left to the discretion of food companies. Objective evaluations suggest that most voluntary reformulation programs have so far resulted in inconsistent levels of compliance across food companies, undermining the effectiveness of these initiatives [9].

Dietary and population-based modeling is an approach regularly used to inform the design and to motivate government decision-makers to implement policy measures to improve public health. Analogous modeling that could attribute potential health gains from private sector actions could similarly inform and drive better business practices. To the best of our knowledge, such modeling has not previously been undertaken with regard to food companies complying with nutrient reformulation programs in the Australian population.

In Australia, average daily sodium intake is approximately 4,000 mg in men and 2,900 mg in women, far more than the WHO-recommended limit of 2,000 mg/d [10]. Excess sodium intake is a major contributor to high blood pressure, which affects 1 of every 3 (34%) adults in Australia. In 2015, the Australian Government established the Healthy Food Partnership (HFP) to engage the food industry to improve diets by identifying priority nutrients, food categories, and setting measurable nutrient targets for food reformulation [11]. In 2020, sodium targets were released for 27 food categories with a 4-year time frame for food companies to voluntarily achieve through reformulation [12]. Preliminary analyses showed that Australia’s sodium targets cover substantially fewer products compared to similar programs already in place in the United Kingdom [13]. It was unknown what the potential health gains of food companies achieving Australia’s sodium targets were and what the likely additional health gains could be if more comprehensive and stricter targets had been adopted instead. Thus, the aim of this study was to estimate (1) the number of deaths, new cases of disease, and disability-adjusted life years (DALYs) that could potentially be averted in Australia if the sodium reformulation targets would be implemented with full compliance; (2) the additional health benefits that could be gained if more comprehensive and more stringent reformulation targets were adopted; and (3) the potential health impact of individual food companies if they were to comply with Australia’s reformulation targets.

## Methods

This project was approved by The University of New South Wales Human Research Ethics Committee (approval number HC 201010).

### Study design

Comparative risk assessment models were used to estimate the number of deaths, new cases of disease, and DALYs that could be averted by (1) the Australian government’s sodium reformulation program [12]; (2) the UK’s sodium reformulation program [14]; and (3) a hypothetical “optimistic” program with more stringent (compared to the UK and Australia) but plausible reformulation targets. The models were used to quantify the theoretical maximal health impact that sodium reformulation programs targeting packaged foods could have in Australia overall, for individual food companies, and for individual food categories targeted.

**Fig 1:**
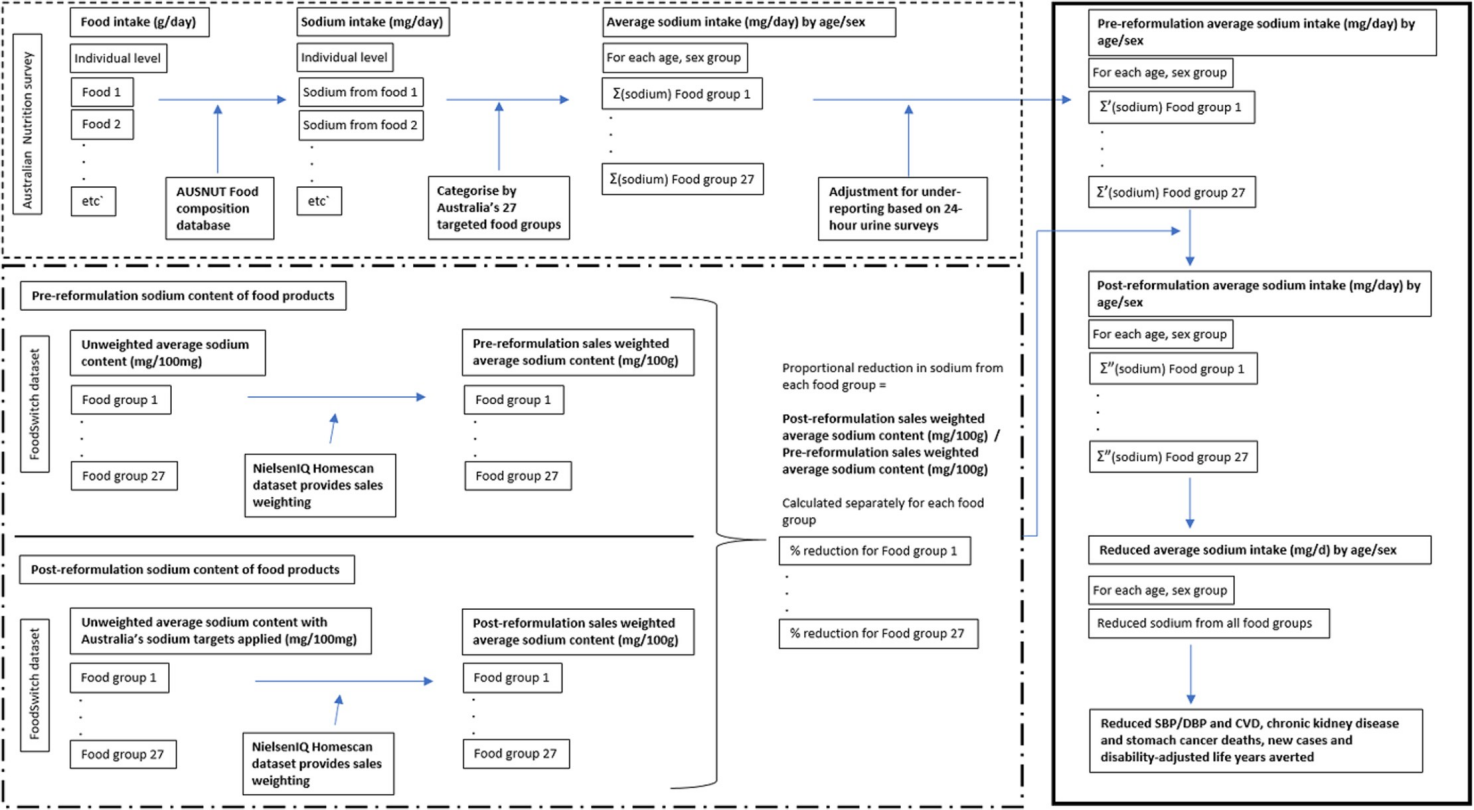
Conceptual model used to estimate the pre-reformulation sodium intake for specific food groups for Australian adults, the post-reformulation sodium intake for specific food groups if companies comply with the Australian government’s 2020 sodium reformulation program (and more stringent programs using the same methods), and the subsequent health benefits of reduced sodium intake for 24 age–sex groups. In the first part of the model (shown in the dashed box), using an Australian nationally representative dietary survey, individual-level food intake data (g/d) at the most specific food level was obtained and matched with the Australian (AUSNUT) food composition database to calculate sodium from each food. Each food and their equivalent sodium contribution were then categorized into the 27 Australian sodium reformulation food groups or 76 UK food groups with sodium targets, and the average sodium intake (mg/d) from all food groups were calculated for each of the 24 age–sex groups. Given that 24-hour diet recalls underestimate food and sodium intake, sodium intake calculated from the diet recall was adjusted up to the daily sodium intake from nondiscretionary sources estimated based on 24-hour urinary sodium excretion for Australian men and women separately. This resulted in the pre-reformulation average sodium overall and for each food group (mg/d) for each age–sex group. To calculate the reduction in sodium intake from each food group as a result of full compliance to the Australian or UK targets, as shown in the dash-dot box, we calculated the pre-reformulation sales-weighted average sodium content of targeted food groups by weighting the sodium content of food products within their food group by their volume of sales (kg). Similarly, the post-reformulation sales-weighted average sodium content was calculated by simulating reductions in the sodium content of foods that were above the target to the targeted level (products with sodium content below the target remained the same). The proportional reduction in sodium from each food group was calculated by dividing the post-reformulation with the pre-reformulation sales-weighted average sodium contents. Finally, we multiplied the pre-reformulation sodium intake from each food group by the proportional reduction in sodium from each food group to obtain the post-reformulation sodium intake from the specific food category (shown in the solid box). The reductions between the pre- and post-reformulation sodium intake for each food group were summed to determine the total average sodium intake reduced for each age–sex group. Using the absolute reduction in average sodium intake for each age–sex group, we estimated the reduction in blood pressure and CVD, CKD, and stomach cancer deaths and new cases averted. Σ represents the sum of multiple items. CKD, chronic kidney disease; CVD, cardiovascular disease; DBP, diastolic blood pressure; SBP, systolic blood pressure.

The datasets used are described in detail in **Table 1,** and the detailed modeling steps are in Text A in S1 File. In brief, the modeling approach involved 4 stages (**Fig 1)**, (1) estimating pre-reformulation sodium intakes from each packaged food group with a sodium target using nationally representative 24-hour dietary recall data of the Australian population (National Nutrition and Physical Activity Survey (NNPAS) and Food Standards Australia New Zealand Food Composition Database) for each age–sex group [15], adjusted for underreporting using sex-specific 24-hour urinary sodium excretion estimates of nondiscretionary sodium intake (Table A in S1 File) [10]; (2) using a nationally representative household grocery shopping panel data (NielsenIQ Homescan [16]) and a brand-specific food composition database (FoodSwitch) [17] to estimate the pre- and post-reformulation sales-weighted average sodium content, and the expected percentage reduction in sales-weighted average sodium content for each targeted food group; (3) estimating the reduction in sodium intake from each individual targeted food group or company based on steps (1) and (2), i.e., multiply prereformulation sodium intake in each food group by the estimated percentage reduction in sales-weighted average sodium content following reformulation (reductions are then summed across food groups to estimate overall reductions in sodium intake); and (4) estimating the effect of reduced sodium intake on CVD, CKD, and stomach cancer outcomes via comparative risk assessment analysis for each age–sex group. Table B in S1 File outlines the model assumptions and restrictions.

**Table 1:** Input data for comparative risk assessment on effect of sodium reformulation targets on death and disease burden in Australia.

Data	Measurement	Source	Use
**Pre-reformulation sodium intake from foods**			
Food intake (g/d)	One 24-hour diet recall from a nationally representative sample	Australian Bureau of Statistics National Nutrition and Physical Activity Survey (2011–2012) [15]	Calculate pre-reformulation weighted mean sodium intake for each targeted food group across each of the 24 age–sex groups. Weights provided by the NNPAS accounted for the probability of selection; the distribution of age, sex, and area of residence of the Australian population; and seasonal adjustment [15,46].
Sodium content of foods (mg/100 g)	Nutrition information of packaged food labels in 2011–2013, based on chemical analyses, recipe calculations, and imputed data	Food Standards Australia New Zealand food composition database (2011–2013) [15]	
Mean sodium intake for adult men and women based on 24-hour urinary sodium excretion	Systematic review and meta-analysis of Australian salt intake measured through 24-hour urinary sodium excretion	Land et al. (2018) Salt consumption by Australian adults: a systematic review and meta-analysis [10]	Adjust sodium intake estimated from the diet recall to 85% of 24-hour urinary sodium excretion. Of 24-hour urinary sodium excretion, 85% was used as an estimate of sodium intake from foods and beverages, as the other 15% comes from discretionary salt (added during cooking or at the table) use [27–29].
**Reduced sodium intake from foods following reformulation**			
Sodium content of brand-specific foods and beverages (mg/100 g)	Nutrition information panels on packaged foods from 5 major supermarkets in Australia in 2018	FoodSwitch (branded food composition database) data (2018) [17]	Calculate pre- and post-reformulation sales-weighted average sodium content of food categories in 2018, under the 3 scenarios (Australian government HFP sodium targets [12], UK sodium targets [14], and plausible sodium targets)
Projected volume (kg) of products purchased in a year	Nationally representative households scan all household grocery purchases over 1 year	NielsenIQ Homescan (2018) [49]	
**Change in sodium intake to disease events, mortality, and DALYs**			
Estimates of mean blood pressure for different age–sex groups in Australia	Measured BP	Australian Bureau of Statistics National Health Survey (2017–2018) [50]	Pre-reformulation estimates of BP for different age–sex groups
Hypertension prevalence	Measured SBP ≥140 mm Hg, diastolic BP ≥90 mm Hg, or receiving medication for high BP	Australian Bureau of Statistics National Health Survey (2017–2018) [50]	Change in BP resulting from sodium reduction varies based on hypertension status
SBP reduction for every 100 mmol sodium reduction	Meta-analysis of randomized trials of the dose response relationship between sodium and BP reduction	Mozaffarian et al (2014) Global sodium consumption and death from cardiovascular causes [22]	Estimate reduction in BP as a result of reductions in mean sodium intake
Relative risk of SBP with CVD outcomes	Pooled cohort studies and meta-analysis of trials	Forouzanfar et al (2017) Global Burden of Hypertension and Systolic Blood Pressure of at Least 110 to 115 mm Hg, 1990–2015 [21] Singh et al (2013) The age-specific quantitative effects of metabolic risk factors on cardiovascular diseases and diabetes: a pooled analysis [20]	Estimate the reduction in the risk of CVD outcomes based on reduction in BP
Relative risk of SBP with CKD	Meta-analysis of large prospective studies	The Global Burden of Metabolic Risk Factors for Chronic Diseases Collaboration (2014) Cardiovascular disease, chronic kidney disease, and diabetes mortality burden of cardiometabolic risk factors from 1980 to 2010: a comparative risk assessment [51]	Estimate the reduction in the risk of CKD outcomes based on reduction in BP
Relative risk of sodium intake on stomach cancer	Meta-analyses of prospective observational studies	GBD 2017 Diet Collaborators (2019) Health effects of dietary risks in 195 countries, 1990–2017: a systematic analysis for the Global Burden of Disease Study 2017 [3,24]	Estimate the reduction in the risk of stomach cancer based on reduction in sodium intake
Estimates and uncertainties of CVD events in Australia	2017 estimates	Global Burden of Disease Study	Comparative risk assessment modelling data input

BP, blood pressure; CKD, chronic kidney disease; CVD, cardiovascular disease; DALY, disability-adjusted life year; GBD, Global Burden of Disease; HFP, Healthy Food Partnership; NNPAS, National Nutrition and Physical Activity Survey; SBP, systolic blood pressure.

### Intervention

The maximum potential health impacts of full compliance to 3 different reformulation programs were modeled. The first was the Australian government’s sodium reformulation program [12], which set maximum sodium content targets for 27 food groups across 12 major product categories (Table C in S1 File) [12]. While the Australian sodium reformulation program is voluntary, we modeled 100% compliance to the sodium targets to estimate the maximum potential impact of such an intervention in Australia.

The second reformulation program modeled was the UK government’s voluntary sodium reformulation targets for 2017 that were released in 2014. Sodium content targets were set for 76 food groups across 27 major product categories (Table D in S1 File) [14]. In 2018, the UK government’s evaluation found 81% of packaged food products from retailers and manufacturers complied with the maximum sodium targets, demonstrating its feasibility [18]. Therefore, we chose to model the UK’s reformulation program in Australia to understand the potential impact of a more comprehensive reformulation program targeting a boarder range of packaged food products, and because the similarity in the UK and Australian food supply means the reformulation program would be feasible for Australia.

The third reformulation program modeled were hypothetical optimistic sodium targets for Australia based on the sales-weighted sodium content (in Australia in 2018) for the 76 food groups targeted by the UK sodium reformulation program. The sodium reformulation targets were defined as the sales-weighted 25th percentile sodium content (mg/100 g) within that category—i.e., 25% of the volume (kg) of foods purchased in each food group already have sodium content that are equal to or below this level, suggesting that it is technically feasible in the current Australian context. These optimistic sodium targets represent an ambitious and yet feasible sodium reformulation program, i.e., having the comprehensive scope of the UK sodium reformulation program plus setting more stringent sodium targets.

### Statistical analysis

#### Comparative risk assessment analysis

Within each sex-and-age-stratum and for each scenario, we calculated the potential impact fraction (PIF) of reformulation for 8 CVD subtypes (ischemic heart disease, ischemic stroke, hemorrhagic stroke, aortic aneurysm, endocarditis, hypertensive heart disease, rheumatic heart disease, and other CVD), CKD, and stomach cancer (detailed in Text B in S1 File). Briefly, the PIF for outcome (o) in age group (a) and sex (s) was calculated as [19]:

$${PIF}_{oas}=\frac{\int_{x=0}^{m}{RR}_{oa}(x)P_{as}(x)dx-\int_{x=0}^{m}{RR}_{oa}(x){P\prime }_{\;as}(x)dx}{\int_{x=0}^{m}{RR}_{oa}(x)P_{as}(x)dx}$$

*P*_*as*_*(x)* and *P’*_*as*_*(x)* are the pre-intervention and post-intervention sodium intake distributions in age group (a) and sex (s). *RR*_*oa*_*(x)* is the relative risk as a function of mmol/d sodium intake (*x*), specific for outcome (*o*) and age. For CVD and CKD, the *RR*_*oa*_*(x)* is defined as:

$${RR}_{oa}(x)=\left\{ \begin{array}{l} e^{\left({lnRR}_{oa}(y)∙\frac{k_{as}}{10}∙\frac{x-TMREL}{100}\right)},x\geq TMREL\\ \;1,x<TMREL \end{array}\right.$$

where lnRR_oa_(y) is the increase in the natural logarithm of the relative risk of outcome (o) in age (a) per 10 mm Hg systolic blood pressure (SBP) increase (Table E in S1 File), derived from previous meta-analyses [20,21]. The k_as_ is the SBP effect estimate of sodium reduction in age group a and sex s, adjusted for hypertension prevalence (measured high blood pressure, i.e., SBP ≥140 mm Hg and/or diastolic BP ≥90 mm Hg) and derived from a previous multivariable-adjusted meta-regression of 103 sodium reduction trials [22]. TMREL is the theoretical-minimum-risk exposure level (i.e., 2.0 ± 0.2 g/d sodium in the primary analysis) [23].

The *RR*_*a*_*(x)* for stomach cancer is defined as:

$${RR}_{a}(x)=\left\{ \begin{array}{l} e^{\left(\frac{{lnRR}_{a}{(x)M}_{Na}(x-TMREL)}{1000}\right)},x\geq TMREL\\ \;1,x<TMREL \end{array}\right.$$

where lnRR_a_(x) is the increase in the natural logarithm of the relative risk of stomach cancer in age (a) per 1 g/d increase of dietary sodium (Table E in S1 File) [24], and M_Na_ is the molar mass of sodium (i.e., 22.99 g/mol).

We utilized estimates and uncertainties of number of disease events (i.e., deaths, incidences, or DALYs) in Australia during 2017 from the Global Burden of Disease Study (Tables F, G, and H in S1 File). The averted number of new cases of CVD, CKD, and stomach cancer was computed by multiplying an age-, sex-, and cause-specific PIF by the current (pre-reformulation) number of events for the same stratum [19,25,26]. The total numbers of new cases averted were calculated as the sum of estimates over all strata, and we summed subtype-specific estimates to generate estimates for total CVD. We excluded inflammatory heart diseases from the estimations of new cases and DALYs, because these diseases develop independently of blood pressure elevation; however, these conditions were included in the estimation of mortality because lower blood pressure will benefit and reduce heart failure mortality in those individuals [20]. For each intervention, we also estimated new cases averted stratified by age (<70 years or ≥70 years) and sex (men and women).

We estimated the incremental number of averted deaths of the UK targets or the optimistic reformulation scenario compared to the Australian government’s targets. For the Australian and UK government targets, we also calculated the number of total averted deaths from CVD, CKD, and stomach cancer for each of the major targeted food categories. We also identified the top 5 companies with the greatest potential to reduce deaths through achieving full compliance with the Australian government’s targets. Based on data usage agreements with NielsenIQ, for the purposes of this analysis, food companies were deidentified; however, we classified whether a company was a “retailer” or a “manufacturer.” Manufacturers were classified as food companies that manufacture and distribute items (also known as “branded products”) for general trade. Retailers were defined as supermarkets that sell their own “private-label” products in their own stores [16], and the estimates for retailers were based on reformulation of their “private-label” products.

#### Uncertainty analysis

The parameter uncertainty in all modeled estimates was quantified using Monte Carlo simulations (*n =* 1,000) [23]. For each simulation, a draw was made from the distributions of (a) pre-reformulation mean sodium intake and the reformulation effect (i.e., change in mean sodium intake post-reformulation) for the specific stratum (Table A in S1 File); (b) hypertension prevalence in each stratum (Table A in S1 File); (c) the main, age-interaction, and hypertension-interaction effects of sodium reduction on SBP; (d) the relative risk of SBP on each CVD and CKD outcome; (e) the relative risk of sodium intake on stomach cancer; (f) the TMREL of sodium; and (g) the current number of events (e.g., deaths) for each outcome. Each set of draws were used to calculate PIF and averted events of each outcome for each age–sex stratum. We reported the 50th and 2.5 to 97.5th percentiles of estimates across all simulations as the central estimate and 95% uncertainty intervals (UIs), respectively.

#### Sensitivity analyses

To evaluate the robustness of our model, we conducted several deterministic sensitivity analyses by changing key model assumptions and inputs (Table I in S1 File). We explored the impact of using lower or higher TMRELs (1.0 ± 0.2 or 3.0 ± 0.6 g/d, respectively) [23]. We also evaluated the impact of lower pre-intervention sodium intakes by assuming that current (pre-reformulation) sodium intakes are 10% lower than estimated in the primary model. Lastly, in order to test the effect of a potentially lower amount of sodium intake attributable to nondiscretionary sources (sodium from all processed and packaged foods and beverages, rather than salt added during cooking or eating), the proportion of sodium estimated to come from such sources was reduced to 75% of total sodium intakes (compared with 85% in the primary model) [27–29].

## Results

### Effects of the reformulation programs on sodium consumption in Australia

If all food companies were to reformulate their products to comply with the Australian government’s sodium targets, sodium intake was estimated to reduce by an average of about 107 mg/d (3.7% of current sodium intake from nondiscretionary sources, SD = 21 mg/d) from a baseline sodium intake of 2,908 mg/d to 2,801 mg/d from nondiscretionary sources (Table J in S1 File). The estimated average sodium reduction was slightly greater for men (mean: 126 mg/d) compared with women (mean: 88 mg/d) and comparable for those ≥70 years (mean: 100 mg/d) and <70 years (mean: 108 mg/d).

If all food companies were to reformulate their products to the UK sodium targets or in line with the optimistic reformulation scenario (25th sales-weighted sodium content in each food category), the reduction in sodium intake was estimated to be approximately double and triple compared to the Australian government’s targets, i.e., decrease on average by 221 mg/d (7.7% of current sodium intake from nondiscretionary sources, SD = 44 mg/d) and 322 mg/d (11.2%, SD = 64 mg/d), respectively (Table J in S1 File).

### Estimated effects on cardiovascular disease, chronic kidney disease, and stomach cancer

**Fig 2:**
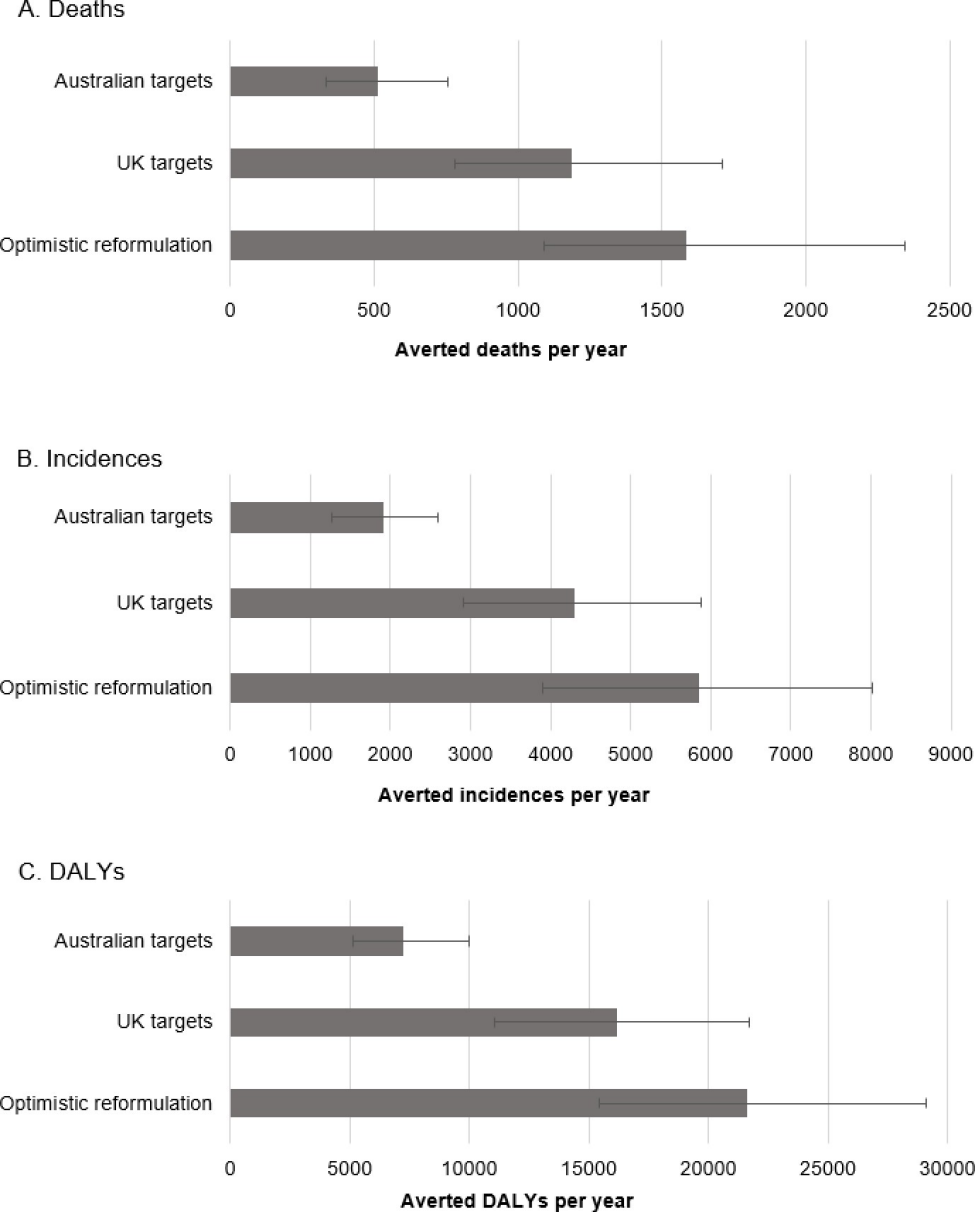
**Total number averted deaths (A), incidences (B), and DALYs (C) per year estimated by intervention.** The error bars indicate the 95% UIs. The reformulation targets for the optimistic reformulation program was defined in each of 76 food groups targeted by the UK reformulation program as the sales-weighted 25th percentile of sodium content in that specific food group. HFP, Australian government’s Healthy Food Partnership sodium reformulation targets. DALY, disability-adjusted life year; UI, uncertainty interval.

**Fig 3:**
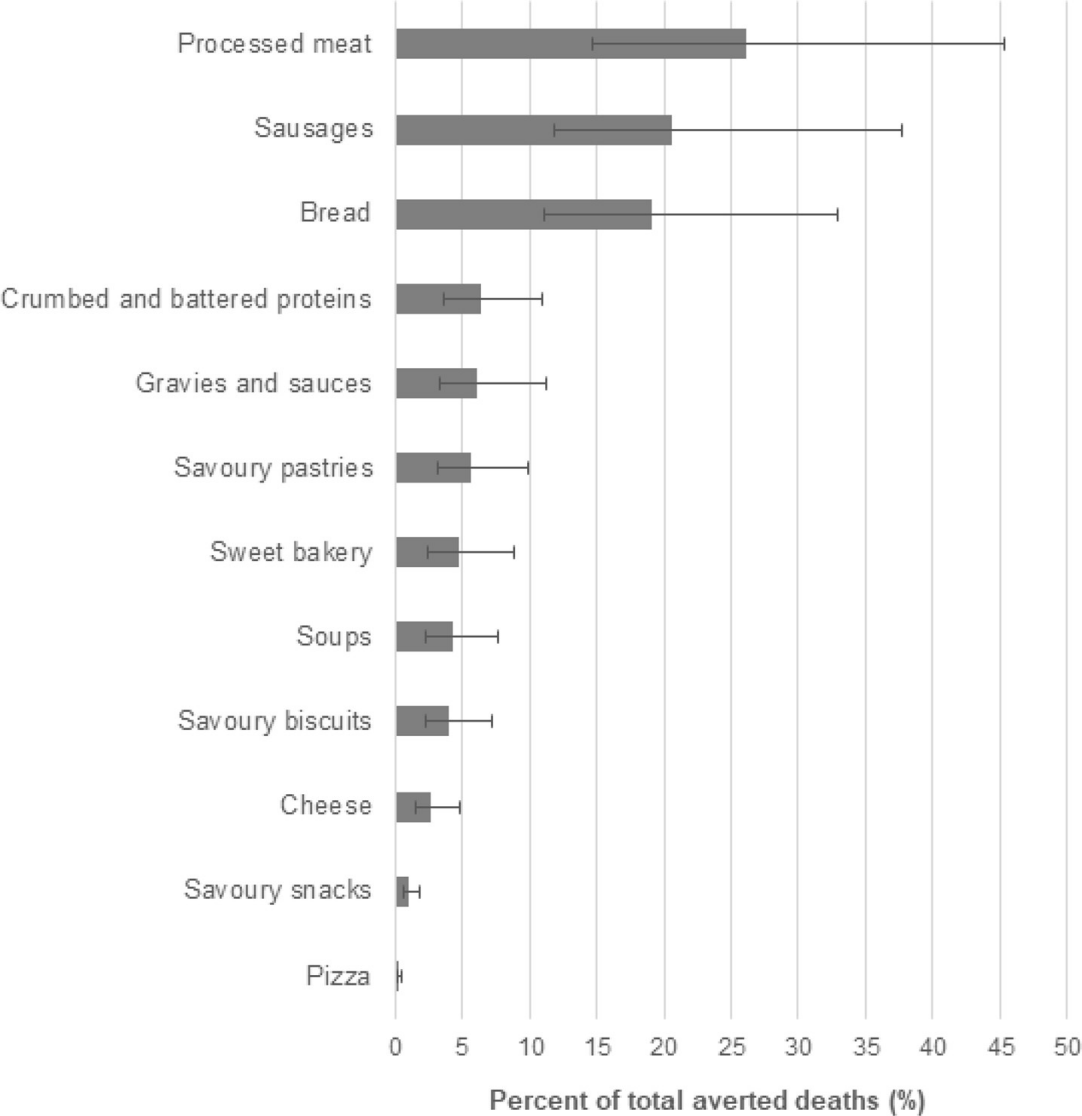
The proportion of deaths from CVD, CKD, and stomach cancer averted each year by food category if the Australian government’s current sodium reformulation targets were achieved. Error bars indicate 95% UIs. Food categories with greatest potential to prevent all-cause death include processed meat (*n =* 134 averted deaths), sausages (*n* = 107), and bread (*n* = 98). CKD, chronic kidney disease; CVD, cardiovascular disease; UI, uncertainty interval.

We estimated that full compliance to the Australian government’s reformulation program could prevent in total approximately 510 deaths per year (95% UI, 335 to 757) **(Fig 2)**. By disease, the number of averted deaths were estimated to be about 420 (262, 641) for CVD (corresponding to about 0.8% of all CVD deaths in Australia during 2017), 50 (29, 91) for stomach cancer (2.8% of all annual deaths from this cause), and 40 (23, 61) for CKD (0.9% of all annual CKD deaths) **(Table 2).** Averted deaths accrued mainly to older adults (≥70 years, 82.3% of total averted deaths) and men (62.1% of total averted deaths). This age and sex pattern was consistent across all disease types analyzed (Fig A in S1 File). In terms of new cases, the Australian government’s reformulation program was estimated to avert some 1,250 (805, 1,733) new cases of CVD (0.8% of all annual CVD events), 570 (361, 806) new cases of CKD (0.7% of total annual), and 110 (64, 168) new cases of stomach cancer (2.9% of total annual) each year. Full compliance to the Australian government’s reformulation program was also expected to prevent approximately 7,240 (5,138 to 10,008) DALYs from CVD, CKD, and stomach cancer annually (Table 2). Across the 12 major food categories covered by the reformulation program, averted deaths were estimated to be mostly attributable to sodium reduction in processed meats (26.2% of all averted deaths, 95% UI, 14.7% to 45.4%), followed by sausages at 20.6% (11.8% to 37.6%) and breads at 19.1% (11.1% to 33.0%) **(Fig 3).** The 9 remaining food categories were estimated to each contribute to <1% to 6% of all averted deaths (Fig 3).

**Table 2:** Estimated intervention effects on CVD, CKD, and stomach cancer in the adult Australian population by full compliance to the Australian government’s 2020 sodium reformulation program.

Metric and disease	No. of events averted (95% UI)	% averted^1^ (95% UI)
**Deaths/year**		
Total^2^	514 (335, 757)	0.9 (0.59, 1.33)
CVD	422 (262, 641)	0.84 (0.52, 1.27)
Ischemic heart disease	252 (144, 401)	0.91 (0.53, 1.44)
Stroke	99 (61, 151)	0.8 (0.49, 1.22)
Other^3^	70 (43, 103)	0.67 (0.43, 0.99)
CKD	38 (23, 61)	0.86 (0.52, 1.37)
Stomach cancer	52 (29, 91)	2.75 (1.52, 4.88)
**Incidences** ^4^ **/year**		
Total	1,921 (1,274, 2,600)	0.78 (0.52, 1.06)
CVD	1,250 (805, 1,733)	0.79 (0.51, 1.1)
Ischemic heart disease	597 (374, 848)	0.95 (0.6, 1.34)
Stroke	274 (176, 393)	0.84 (0.54, 1.19)
Other^3^	373 (244, 513)	0.6 (0.4, 0.82)
CKD	565 (361, 806)	0.67 (0.44, 0.96)
Stomach cancer	105 (64, 168)	2.86 (1.76, 4.53)
**DALYs/year**		
Total	7,238 (5,138, 10,008)	0.95 (0.67, 1.31)
CVD	5,714 (3,846, 8,179)	0.87 (0.58, 1.24)
Ischemic heart disease	3,221 (2,158, 4,760)	0.93 (0.61, 1.37)
Stroke	1,491 (985, 2,171)	0.84 (0.56, 1.24)
Other^3^	985 (655, 1,425)	0.73 (0.48, 1.04)
CKD	527 (341, 772)	0.73 (0.47, 1.07)
Stomach cancer	987 (647, 1,483)	2.97 (1.97, 4.48)

^1^% averted refers to the percent of deaths, incidences, and DALYs that would be averted out of the total disease deaths, incidences, and DALYs in 2017.

^2^For each individual and aggregate outcome, the calculation of the events averted is repeated 1,000 times, and the median of the 1,000 estimates is the point estimate reported. Therefore, the sum of the point estimates of individual outcomes may not necessarily equal the point estimate of the aggregate outcomes.

^3^Other CVD: aortic aneurysm, atrial fibrillation and flutter, cardiomyopathy and myocarditis, endocarditis, hypertensive heart disease, miscellaneous CVD, peripheral artery disease, and rheumatic heart disease.

^4^The global burden of disease define incidence as “the number of new cases of a given cause during a given period in a specified population.”

CKD, chronic kidney disease; CVD, cardiovascular disease; DALY, disability-adjusted life year; UI, uncertainty interval.

### Comparison of Australian government program to the UK program and an alternate optimistic reformulation program

Compared to the Australian government’s reformulation targets, full compliance to the UK targets was estimated to avert around 660 more deaths in Australia (95% UI, 207 to 1,227) **(Fig 2,** Table K in S1 File**).** The UK targets could also prevent an additional 2,341 (785 to 4,046) new cases of CVD, CKD and stomach cancer annually and an additional 8,748 (3,109 to 15,552) DALYs each year (Table K in S1 File).

If Australia had instead adopted more stringent targets such as the “optimistic reformulation” scenario (based on sales-weighted 25th percentile sodium content of the UK targeted food categories), this was estimated to prevent about 1,065 more deaths (511 to 1,856), around 3,929 (1,907 to 6,098) more new cases, and some 14,435 (7,538 to 22,143) more DALYs per year compared to the Australian government’s targets (Fig 2, Table K in S1 File).

All 12 major food categories covered by the Australian government’s program were also targeted by the UK reformulation program. Similar to the Australian government’s reformulation program, the proportion of averted deaths was estimated to be largest for the meat category (43.6%, 95% UI, 24.3% to 75.6%), followed by bread at 11.9% (6.8% to 20.4%) and cheese at 8.1% (4.4% to 15.1%) (Fig B in S1 File). The UK reformulation program has set targets for additional categories not targeted by the Australian government’s program. These include ready meals and meal centers, which accounted for 4.0% of averted deaths (2.3% to 6.9%), baked beans at 3.2% (1.8% to 5.8%), and canned vegetables at 2.5% (1.5% to 4.5%) (Fig B in S1 File).

### Intervention effects on cardiovascular disease, chronic kidney disease, and stomach cancer by food company

The NielsenIQ database identified over 300 larger food companies that sold products covered by the Australian government’s sodium reformulation targets. We estimated that over half (59%) of deaths that could be averted through compliance with the Australian government’s reformulation program would be attributed to 5 food companies **(Table 3).** Each of these companies were estimated to account for between approximately 5.8% to 18.5% of averted deaths, largely due to reformulation in the food categories processed meat, bread, crumbed and battered proteins (e.g., chicken nuggets), and sausages. Of these 5 companies, the top 3 were all grocery retailers, i.e., the averted deaths would be attributable to reformulation of their private-label products (Table 3).

**Table 3:** Total averted deaths per year across top 5 companies with greatest potential to reduce death from full compliance with the Australian government’s 2020 sodium reformulation targets.

Company rank and manufacturer type	Total averted deaths	
	N (%)	Top 3 food categories with the greatest impact if full compliance to the Australia sodium targets were achieved^1^
1. Retailer	95 (18.5)	Processed meat, bread, crumbed and battered proteins
2. Retailer	94 (18.2)	Sausages, bread, crumbed and battered proteins
3. Retailer	47 (9.1)	Bread, processed meat, sausages
4. Manufacturer	39 (7.5)	Processed meat, crumbed and battered proteins, sausages
5. Manufacturer	30 (5.8)	Bread, processed meat, savory biscuits
Others	209 (40.7)	

^**1**^The categories listed are the top 3 food categories within each food company that have the greatest potential to reduce sodium intakes and therefore contribute to deaths averted.

### Sensitivity analyses

Across the 4 different sensitivity analysis scenarios, modeled estimates of deaths averted through full compliance with the Australian government’s reformulation program differed by −19% to +13% of the primary model estimate. The greatest impact on the modeled estimate was when assuming a TMREL of 1.0 ± 0.2 g sodium/d, which resulted in 575 averted deaths per year (95% UI, 375 to 839) (Fig C in S1 File). On the contrary, when assuming a higher TMREL value of 3.0 ± 0.6 g sodium/d, the number of averted deaths reduced to 415 (272 to 637). Other sensitivity analysis scenarios, including assuming a 10% lower pre-reformulation sodium intake and lower proportion of sodium from nondiscretionary sources (processed and packaged food and foods consumed outside of the home), had relatively minor impact on the modeled estimates compared to the primary model (Fig C in S1 File).

## Discussion

Based on the current modeling analyses, the Australian government’s HFP sodium reformulation program could reduce average population sodium intake (from nondiscretionary sources) by up to 3.7% and prevent around 514 deaths and 1,921 new cases of CVD, CKD, and stomach cancer in Australia per year. The majority of these benefits could be achieved if a handful of major food companies were to fully comply with the reformulation targets. The implementation of more comprehensive sodium targets such as those set by the UK government and our proposed optimistic targets were estimated to more than double and triple the number of deaths averted, respectively.

The relatively limited impact of the Australian HFP sodium targets on sodium consumption and health can be explained jointly by its low product coverage coupled with lenient sodium content targets. Our analyses identified several key food products (i.e., butter, spreads, ready meals, table sauces, canned fish, and breakfast cereals) contributing to sodium consumption in Australia that do not have targets but are covered by the UK reformulation program. Furthermore, a large proportion of some targeted products (e.g., 93% of plain corn and rice cakes, 71% of cheddar style cheeses, 66% of plain biscuits, and 66% of sweet bakery) already comply with the Australian government’s sodium targets, indicating the targets are too lenient [13]. The optimistic scenario demonstrates that through feasible increases in the product coverage of targets (to the level adopted in the UK), and tightening of the sodium targets (to the level where a quarter of existing products within each category already comply in Australia), sodium reformulation programs can have a substantial health impact in Australia.

The 5-year planning and development process that has defined the Australian government’s HFP sodium reformulation program [12] appears to represent a significant missed opportunity to save lives in Australia. The Australian government could have simply adopted the UK government’s sodium reformulation targets, leveraging the widespread industry and public health consultation done in a market with a directly comparable packaged food supply, and real-world validation of its feasibility (shown by over 80% compliance with the targets achieved in 2018) [18]. This would almost certainly have been time-saving and cost-saving and would have delivered a more powerful program able to avert 1,174 premature deaths each year, which is comparable to the toll from road deaths in Australia in 2019 [30]. To accelerate the implementation of sodium reformulation programs, governments worldwide should consider examining existing sodium reformulation targets and adopting strict sodium targets for a comprehensive range of food categories contributing to sodium in their local diet. WHO’s recently established sodium benchmarks, which cover 58 food categories and were selected based on the lowest sodium values from existing targets and thorough technical consultation, represent the gold standard, and a clear starting point for governments considering to implement sodium reformulation targets [31].

The health gains estimated for the Australian government’s sodium targets are highly optimistic, since previous experience suggests that under a voluntary reformulation scheme, not all companies will fully comply with the suggested sodium targets. For instance, only 67% of bread products and 47% of processed meat products met a prior set of voluntary sodium targets set by the Australian Food and Health Dialogue (the predecessor to the HFP), by the 2013 deadline [32]. Given the already limited projected impact of the Australian government’s HFP sodium reformulation program under a full implementation scenario, Australian government’s actions to achieve widespread compliance by industry will be vital to achieving health impact. These should include robust monitoring and evaluation efforts [33,34] and media engagement to profile over- and underachieving companies [33,35,36], and if voluntary efforts fail to achieve meaningful reductions, mandatory targets with penalties for noncompliance should be considered [37]. The threat of mandatory targets was also used by the UK government with great effect [33]. Furthermore, given that all 3 reformulation scenarios could not lower sodium intake levels to the WHO-recommended level of <2,000 mg/d, the reformulation program should be considered as part of a coordinated food and nutrition strategy that addresses not only other sources of sodium intake (discretionary salt use and meals consumed out of home such as from takeaway outlets, cafes, and restaurants) but also healthier dietary patterns such as the replacement of processed foods for fresh produce [8,35,38].

To our knowledge, our study is the first to attribute the potential health impact that individual food companies can make through sodium reformulation. Our results highlight the outsized health impact that the retailers and a few major manufacturers could have by reformulating their product portfolios. In Australia, the food retail landscape is dominated by a small number of companies and the market share of their private-label products has grown rapidly over the last decade [39]. While it would be complex to implement mandatory sodium targets only for a handful of food companies and may have unintended consequences on competitive balance, the findings highlight the opportunity the Australian government can have to deliver a highly focused engagement and enforcement program at low cost for high impact. For instance, the provision of comparative nutrient information appears to encourage sodium reformulation among the retailers’ private-label products [40], and effects may be enhanced by serial monitoring and reporting of progress against targets.

### Strengths

Our study has several strengths. Nationally representative data (national nutrition survey (NNPAS) [15], NielsenIQ Homescan, and blood pressure data) were used whenever possible, increasing the generalizability and validity of the results. Several steps were undertaken to precisely and realistically estimate sodium consumption before and after reformulation from each of the targeted food categories and individual food companies. First, we utilized dietary intake data from a nationally representative survey to accurately categorize foods according to the Australian government and UK targeted food group criteria (i.e., allowing us to distinguish foods made from scratch and foods prepared from restaurants and takeaway that are not covered by the Australian government or UK reformulation targets). Second, we accounted for underestimations of sodium intake when measured through dietary surveys by adjusting to the precisely estimated levels based on 24-hour urine collections [10]. Third, we used product-specific sodium content (rather than generic sodium contents for food categories) and weighted products by sales data (NielsenIQ Homescan) [16], which is vital to account for the large variations in sodium content among products within the same food category [41], and the different market share of products. Both steps are rarely done in previous studies [42–44], including the dietary impact modeling that was conducted to inform the Australian government’s sodium targets due to a lack of sales data [45]. Fourth, we simulated changes in the sodium content post-reformulation at the food product level, which allowed us to accurately attribute deaths averted to individual food categories and companies. Furthermore, we estimated the effects of the reformulation programs for each of the 24 age–sex groups to account for differences in dietary intake; the dose–response relation between sodium and blood pressure; blood pressure distribution; and relative risk of CVD, CKD, and stomach cancer by age and sex groups. Finally, we conducted several sensitivity analyses to assess the influence of varying inputs and assumptions, which indicated the robustness of our findings.

### Limitations

Our study also has some limitations that should be considered. The national nutrition survey used to estimate food consumption, and the dietary sources of sodium were conducted in 2011 to 2012, and were based on a single 24-hour diet recall on any given day, and therefore may not reflect usual sodium intake (e.g., estimated by the NCI method) [15,46]. However, these sodium intake estimates were closely aligned with the 2019 Global Burden of Disease estimates [47], are from the most current nationally representative nutrition survey in Australia, and there is some evidence to suggest that there have been no substantial changes in sodium consumption over the last few years [10,27]. While we adjusted the total sodium intake estimated by the dietary surveys up to 24-hour urinary sodium to account for underreporting, this was equally applied across all food groups. In reality, some food groups (e.g., unhealthy foods) are subject to greater underreporting than others. In addition, we used nationally representative sales data from NielsenIQ Homescan to calculate the sales-weighted average sodium content in targeted food groups for all age and sex groups, though the market share for certain brands or products may differ by age or sex groups. We did not account for possible changes in population choices of foods after reformulation; however, previous experience suggests that food companies can make gradual reductions in sodium intake without consumers noticing [33]. While we modeled full compliance with the Australian government and UK sodium reformulation targets, under a voluntary system, there is uncertainty about which food manufacturers will choose to comply with the targets, which products will be reformulated, and the extent of reformulation. It should also be noted that we modeled the impact of the UK’s maximum (rather than average) sodium targets as it is more comparable to Australia’s existing sodium reformulation program, which only include maximum targets, and it is likely to provide a more conservative estimate of the health impact of the UK’s sodium reformulation program.

Comparative risk assessments provide a simple tool for evaluating the population health impact of modifying the population distribution of exposure to the risk factor (sodium intake) using consistent and comparable methods. However, the weaknesses, compared to more complex modeling approaches, should be considered. This includes the absence of a time dimension (such as the time lag between the intervention and disease outcomes), inability to account for recurring events, and inability to model the interactions between individuals, populations, or their environments. We did not evaluate the potential impact on health equality; however, previous studies suggest that disadvantaged groups may have greater reductions in sodium intake and, therefore, more health benefits from sodium reformulation programs [13,48]. In addition, estimates of disease burden pre-reformulation were based on the Global Burden of Disease study, and while the methodology behind these estimates is continuously improving, estimates of nonfatal outcomes may be less accurate [20–22]. It should be noted that our estimates of the health benefits of sodium reduction does not include other potential health outcomes (e.g., the direct risk of sodium intake on CVD and CKD outcomes) that are not mediated by blood pressure except for stomach cancer, and for the uncertainty analysis, we independently drew random samples from distributions of input variables that may be correlated. However, these methods are consistent with previous sodium reduction modeling studies [3,22,23]. Our modeling study does not prove that a sodium-reducing food reformulation programs will prevent morbidity and mortality; rather, it provides important quantitative estimates, corresponding uncertainty, and assessments of the sensitivity of the findings to different inputs, to guide the design, implementation, and evaluation of an appropriate reformulation policy.

## Conclusions

The sodium reformulation targets set by the Australian government’s HFP in 2020 were estimated to result in approximately 500 deaths averted if food manufacturers achieved full compliance. The adoption of more comprehensive sodium targets such as those set by the UK government for 2017 could more than double the number of deaths averted. Our results suggest that the Australian government’s sodium reformulation intervention should be expanded to target more food products, adopt more stringent sodium content targets, and be made mandatory, in order to prevent thousands more new cases of CVD, CKD, and stomach cancer annually. Our findings suggest that reformulation of private label products should be prioritized by retailers in Australia to help achieve most of the health gains from the sodium reformulation program.

## Supporting information

S1 File Supporting information, tables, and figures. Text A. Data sources and detailed modeling steps. Table A. Sodium intake from nondiscretionary sources, SBP, and hypertension prevalence (measured high blood pressure) in Australian adults, by age and sex. Table B. Key assumptions and restrictions of models that estimate the impact of reformulation programs on sodium intake and blood pressure. Table C. List of the food categories (and the target levels) included in the Australian government’s HFP’s sodium reformulation program. Table D. List of the food categories (and the target levels) included in the UK sodium reformulation program. Text B. Comparative risk assessment methods. Table E. Relative risk estimates and corresponding 95% confidence intervals of CVD subtypes, CKD, and stomach cancer per 10 mm Hg increase in SBP or 1 g/d increase in sodium intake. Table F. Estimated number of deaths (95% UIs) in Australia 2017 per disease, stratified by age and sex. Table G. Estimated number of incidences (95% UIs) in Australia 2017 per disease, stratified by age and sex. Table H. Estimated number of DALYs (95% UIs) in Australia 2017 per disease, age, and sex. Table I. Model inputs and assumptions in sensitivity analyses. Table J. Estimated sodium reductions from full compliance across each reformulation scenario, stratified by age and sex. Fig A. Estimated effects of full compliance to the Australian government’s sodium targets on CVD, CKD, and stomach cancer mortality by age (<70 years, ≤70 years) and sex (men, women). Values are central estimates (median) of *n =* 1,000 simulations, and error bars indicate 95% UIs. Table K. Estimated intervention effects on CVD, CKD, and stomach cancer in the adult Australian population by the Australian government’s sodium reformulation targets compared with the UK sodium targets and optimistic reformulation scenario. Fig B. The proportion of deaths from CVD, CKD, and stomach cancer averted each year by food category if all foods were reformulated to UK sodium targets. Error bars indicate 95% UIs. Food categories with greatest potential to prevent total deaths include meat products (*n =* 512 averted deaths), bread (*n* = 140), and cheese (*n* = 96). Fig C. Effects of full compliance to the Australian government’s sodium targets on averted deaths from CVD, CKD, and stomach cancer estimated by the primary model and in the sensitivity analyses. Gray bars indicate central estimates of *n* = 1,000 simulations, and error bars represent 95% UIs around the central estimates. Values inside bars indicate the central estimate as a proportion of the central estimate of the primary analysis. The primary model included a TMREL of 1.6–2.4 g/d and assumption that 85% of daily sodium intakes comes from nondiscretionary sources (packaged foods, processed foods, and foods consumed out of home). CKD, chronic kidney disease; CVD, cardiovascular disease; DALY, disability-adjusted life year; HFP, Healthy Food Partnership; SBP, systolic blood pressure; TMREL, theoretical minimum risk exposure level; UI, uncertainty interval.
(DOCX)

## Abbreviations

CKD: chronic kidney disease
CVD: cardiovascular disease
DALY: disability-adjusted life year
HFP: Healthy Food Partnership
NCD: noncommunicable disease
NNPAS: National Nutrition and Physical Activity Survey
PIF: potential impact fraction
RR: relative risk
SBP: systolic blood pressure
TMREL: theoretical minimum risk exposure level
UI: uncertainty interval
WHO: World Health Organization
