# Supplementary material for: The estimated health impact of sodium reduction through food reformulation in Australia: A modeling study
Source: PLoS Med. 2021 Oct 26;18(10):e1003806. doi: 10.1371/journal.pmed.1003806 (PMC8547659; doi:10.1371/journal.pmed.1003806)
Supplement: S1 File — Text A. Data sources and detailed modeling steps. Table A. Sodium intake from nondiscretionary sources, SBP, and hypertension prevalence (measured high blood pressure) in Australian adults, by age and sex. Table B. Key assumptions and restrictions of models that estimate the impact of reformulation programs on sodium intake and blood pressure. Table C. List of the food categories (and the target levels) included in the Australian government’s HFP’s sodium reformulation program. Table D. List of the food categories (and the target levels) included in the UK sodium reformulation program. Text B. Comparative risk assessment methods. Table E. Relative risk estimates and corresponding 95% confidence intervals of CVD subtypes, CKD, and stomach cancer per 10 mm Hg increase in SBP or 1 g/d increase in sodium intake. Table F. Estimated number of deaths (95% UIs) in Australia 2017 per disease, stratified by age and sex. Table G. Estimated number of incidences (95% UIs) in Australia 2017 per disease, stratified by age and sex. Table H. Estimated number of DALYs (95% UIs) in Australia 2017 per disease, age, and sex. Table I. Model inputs and assumptions in sensitivity analyses. Table J. Estimated sodium reductions from full compliance across each reformulation scenario, stratified by age and sex. Fig A. Estimated effects of full compliance to the Australian government’s sodium targets on CVD, CKD, and stomach cancer mortality by age (<70 years, ≤70 years) and sex (men, women). Values are central estimates (median) of n = 1,000 simulations, and error bars indicate 95% UIs. Table K. Estimated intervention effects on CVD, CKD, and stomach cancer in the adult Australian population by the Australian government’s sodium reformulation targets compared with the UK sodium targets and optimistic reformulation scenario. Fig B. The proportion of deaths from CVD, CKD, and stomach cancer averted each year by food category if all foods were reformulated to UK sodium targets. Error bars indica [file pmed.1003806.s001.docx]

**S1 File. Supporting Information, Tables and Figures**

**Text A.** **Data sources and detailed modelling steps**

*Estimation of pre-reformulation sodium intake from targeted food groups and overall*

Sodium intake from packaged foods and beverages (but not foods eaten out-of-home, foods made from scratch, and discretionary salt added by the consumer during cooking or eating) were estimated for adults in 24 age-sex groups using the latest nationally representative 2011-12 National Nutrition and Physical Activity Survey (NNPAS) survey and the corresponding Australian Food Composition Database (AUSNUT 2011-13).[1] We utilised 24-hour diet recall data from adults aged 25 and over, comprising of 8655 participants weighted for the probability of being selected; the distribution of age, sex and area of usual residence of the Australian population; and seasonal adjustment.[1] Packaged foods and beverages were categorised according to the Australian government’s Healthy Food Partnership (HFP) and UK sodium reformulation food group criteria.[2,3] Total daily sodium intake from non-discretionary sources was calculated as the sum across all food groups including those not targeted by the sodium reformulation programs. To account for known under-reporting of food and therefore sodium intake in 24-hour diet recalls, [4,5] the total sodium intake from all food and beverages (not including discretionary salt) estimated from NNPAS was adjusted upwards to the optimally estimated sodium intake based on 24-hour urine excretion for Australian adults.[6] This was done using a sex-specific conversion ratio by 1) taking the estimated usual average sodium intake assessed by 24-hour urine (based on a systematic review and meta-analysis results involving n=3896 Australian adults), multiplied by 0.85 to derive the estimated 85% sodium obtained from non-discretionary sources (processed, packaged food and foods consumed outside of the home),[7-9] 2) dividing the resulting average 24-hour urine estimated sodium from non-discretionary sources by the average NNPAS non-discretionary sources of sodium intake to derive an under-reporting factor, and 3) multiplying the average sodium intake from each food group by the under-reporting factor, i.e. we assumed that the degree of under-reporting is similar across food groups (Table B in S1 File).

*Estimation of counterfactual sodium intake (post-reformulation)*

For each food group targeted by the Australian government’s or UK sodium reformulation, we calculated the sales-weighted average sodium content (mg/100g), i.e. the average sodium content of products (mg/100g) within a food group weighted by their volume of sales (kg), pre and post sodium reformulation in Australia. For this, we utilized two key datasets:

1. NielsenIQ Homescan data – data on household-level food and beverage purchases collected from a nationally representative cohort of approximately 7,188 households from 1 January to 31 December 2018. The NielsenIQ Homescan data provided information about all foods purchased and brought into the home by the Homescan participants, and projected to reflect national household grocery sales.[10]
2. FoodSwitch database – a brand-specific food composition database which systematically collects the nutrition content data from the nutrition information panel (NIP) of packaged foods sold in five major supermarket chains (together accounting for approximately >85% of grocery sales) in Australia including Aldi, Coles, Woolworths, Harris Farms, and IGA in 2018. FoodSwitch data was used to determine the sodium content of packaged foods.[11]

The sales volume (from NielsenIQ Homescan) and sodium content of each food product (FoodSwitch) were linked by the barcode, as previously reported.[10] Once linked, the current (pre-reformulation) sales-weighted average sodium content (mg/100g) was calculated for each of the targeted food categories. Then, we calculated the counterfactual sales-weighted sodium content for each of the three reformulation scenarios:

1. Full (100%) compliance with the Australian government’s HFP sodium targets
2. Full (100%) compliance with the UK sodium targets
3. Reduction to the current 25^th^ sales-weighted percentile sodium content for each of the 80 food categories that make up the UK targets (called ‘optimistic’ reformulation henceforth).

The post-reformulation sales-weighted average sodium content was estimated under the scenario that all targeted foods with current sodium levels exceeding the sodium targets were hypothetically reformulated to the category-specific sodium targets. Whereas products with current sodium content below or at the sodium targets were assumed to retain their existing sodium content. We assumed that reformulation of several products within a food category to the same targeted sodium level, as well as reformulation in several food categories across the food supply would not affect household food purchasing patterns. For each food category, the proportional reduction (%) in sales-weighted average sodium content were calculated from the pre- and post-reformulation sales-weighted average sodium content:

$$Proportional Reduction\left( \% \right)=100 \left( \frac{{Content}_{post}}{{Content}_{pre}} \right)$$

Finally, the current sex-and-age-specific daily sodium intake from each of the targeted food categories were multiplied by the estimated proportional reduction (%) in sales-weighted average sodium content to obtain the sex-and-age-specific sodium intake post-reformulation for each food category. The estimated pre-reformulation sodium intake was subtracted by the post-reformulation sodium intake for each food category to calculate the food-category specific reductions in sodium intake. We summed each targeted food-category specific reduction in sodium intake to calculate the total reduction in daily sodium intake from non-discretionary foods post-reformulation for each sex-and-age specific group. We used a similar approach in order to determine sex-and-age specific reductions in daily sodium intake that could be attributed to individual food companies, i.e. we simulated reductions in sodium content of targeted products belonging to individual companies.

**Table A.** **Sodium intake from non-discretionary sources, systolic blood pressure, and hypertension prevalence (measured high blood pressure) in Australian adults, by age and sex.**

|  | **Mean (SD) sodium intake, g/d^1^** | |  | **Mean systolic blood pressure (95% CI), mmHg^2^** | |  | **Hypertension prevalence (95% CI), %^2^** | |
| --- | --- | --- | --- | --- | --- | --- | --- | --- |
| **Age, y** | **Men** | **Women** |  | **Men** | **Women** |  | **Men** | **Women** |
| 25 to 29 | 3.73 (2.53) | 2.70 (2.58) |  | 120.1 (118.9-121.2) | 108.2 (107.3-109.1) |  | 10.2 (7.6-12.8) | 5.3 (3.9-6.6) |
| 30 to 34 | 3.72 (2.73) | 2.60 (1.93) |  | 120.6 (119.6-121.5) | 109.5 (108.6-110.4) |  | 12.6 (10.3-14.9) | 8.5 (7.0-9.9) |
| 35 to 39 | 3.97 (2.92) | 2.72 (2.01) |  | 121.1 (120.1-122.0) | 111.5 (110.6-112.4) |  | 16.4 (13.8-19.0) | 12.0 (10.3-13.8) |
| 40 to 44 | 3.55 (2.61) | 2.54 (1.88) |  | 122.6 (121.6-123.6) | 114.3 (113.3-115.2) |  | 21.9 (19.1-24.8) | 15.5 (13.4-17.7) |
| 45 to 49 | 3.35 (2.09) | 2.51 (1.86) |  | 125.2 (124.2-126.2) | 117.8 (116.7-118.9) |  | 29.1 (26.0-32.3) | 19.0 (16.4-21.6) |
| 50 to 54 | 3.51 (2.18) | 2.43 (1.83) |  | 128.0 (126.9-129.0) | 121.4 (120.2-122.6) |  | 33.8 (30.7-36.8) | 23.1 (20.3-26.0) |
| 55 to 59 | 3.05 (1.90) | 2.31 (1.74) |  | 130.9 (129.7-132.1) | 125.0 (123.8-126.2) |  | 35.9 (33.4-38.5) | 28.0 (25.0-31.0) |
| 60 to 64 | 2.95 (1.84) | 2.38 (1.79) |  | 133.0 (131.7-134.3) | 128.5 (127.2-129.8) |  | 38.3 (35.7-40.9) | 32.4 (29.3-35.6) |
| 65 to 69 | 2.93 (1.83) | 2.24 (1.69) |  | 134.3 (133.0-135.6) | 131.9 (130.6-133.2) |  | 40.8 (37.6-44.0) | 36.5 (33.2-39.8) |
| 70 to 74 | 2.95 (2.04) | 2.07 (1.35) |  | 135.3 (133.8-136.8) | 134.7 (133.3-136.1) |  | 42.0 (38.2-45.9) | 40.3 (36.4-44.2) |
| 75 to 79 | 2.73 (1.89) | 2.06 (1.35) |  | 136.1 (134.4-137.8) | 136.8 (135.3-138.4) |  | 41.9 (37.4-46.4) | 43.8 (38.9-48.7) |
| 80+ | 2.63 (1.82) | 2.22 (1.46) |  | 138.8 (135.9-141.8) | 139.6 (136.9-142.3) |  | 47.7 (39.6-55.9) | 46.5 (39.2-53.9) |

^1^ Sodium intake from non-discretionary sources (assumed to be 85% of total sodium intake) estimated using data from Australian Bureau of Statistics National Nutrition and Physical Activity Survey (2012-13), Food Standards Australia New Zealand food composition database (2011-13), and Land et al. (2018) Salt consumption by Australian adults: a systematic review and meta-analysis. See page 2, *Current (pre-reformulation) sodium intake from specific food groups and overall for Australian adults,* for details*.*

^2^From Australian Bureau of Statistics National Health Survey (2017-18).

**Table B. Key assumptions and restrictions of models that estimate the impact of reformulation programs on sodium intake and blood pressure**

| **Category** | **Assumption/Restriction** | **Motivation** |
| --- | --- | --- |
| **Sodium intake estimation** | 85% of sodium estimated from 24-hour urinary sodium comes from packaged, processed and foods consumed out of home. | Prior literature analysing sources of dietary salt [7-9]. |
|  | The level of underreporting of food intake is similar across food groups. | Lack of data suggesting differential misreporting of sodium between food categories. |
|  | The level of underreporting is similar across age groups | Lack of data suggesting differential misreporting of sodium between age groups. |
|  | Food and sodium intake in Australia have remained stable since the most recent National nutrition survey (2012-13). | Systematic review and repeated longitudinal assessment of 24-hour urine sodium found no appreciable change in average sodium intake over the past 30 years [6,7]. |
| **Policy effect** | The proportional sodium reduction of each food group is similar in all age-sex groups. | Data from the Homescan panel were based on households of mixed ages and sex, limiting our ability to calculate age-sex specific proportional sodium reduction of different food groups. Hence, the primary analyses were based on the population average market shares within food categories [12]. |
| **Comparative risk assessment** | Rheumatic heart disease, endocarditis, and myocarditis will be included in the modelling of cardiovascular disease mortality but not incident cardiovascular disease or cardiovascular disease -attributed disability-adjusted life years. | Although these cardiovascular disease subtypes are caused by infection or autoimmune responses, high SBP has been associated with an increasing risk of fatal heart failure which is considered an intermediate cause of death [13,14]. |
|  | All beneficial effects on cardiovascular disease and chronic kidney disease from sodium reduction are mediated through blood pressure. | Conservatively, we did not consider potential benefits on cardiovascular health independent of blood pressure (e.g. reduced vascular stiffness and fibrosis from reduced sodium intake) [15]. |
|  | The relationship of blood pressure and cardiovascular disease follows a log-linear dose-response until a systolic blood pressure level of 110 mm Hg. Below which no further lowering of risk was assumed. | Consistent with the approach of the Global Burden of Disease study [16]. |
|  | The reduction in sodium intake and benefits on blood pressure and disease outcomes are assumed to be concurrent. | Consistent with previous studies [13,15]. |

**Table C.** **List of the food categories (and the target levels) included in the Australian government’s Healthy Food Partnership’s sodium reformulation program.**

| **Food category** | **Sub-category** | **Target (mg/100g)** |
| --- | --- | --- |
| Bread | Leavened breads | 380 |
|  | Flat breads | 450 |
| Cheese | Cheddar style cheeses | 710 |
|  | Processed cheeses | 1270 |
| Crumbed and battered proteins | Meat and poultry | 450 |
|  | Seafood | 270 |
| Gravies and sauces | Gravies and finishing sauces | 450 |
|  | Pesto | 720 |
|  | Asian style sauces | 680 |
|  | Other savoury sauces | 360 |
| Pizza | Pizza | 450 |
| Processed meat | Ham | 1005 |
|  | Bacon | 1005 |
|  | Processed deli meat | 720 |
|  | Frankfurts and saveloys | 900 |
| Sausages | Sausages | 540 |
| Savoury biscuits | Plain savoury crackers and biscuits | 630 |
|  | Plain corn, rice and other cakes | 270 |
|  | Flavoured biscuits, crackers and corn cakes | 720 |
| Savoury pastries | Dry pastries | 500 |
|  | Wet pastries | 400 |
| Savoury snacks | Potato snacks | 500 |
|  | Salt and vinegar snacks | 810 |
|  | Extruded and pelleted snacks | 720 |
|  | Vegetable, grain and other snacks | 450 |
| Soups | Soups | 280 |
| Sweet bakery | Cakes, muffins and slices | 360 |

**Table D. List of the food categories (and the target levels) included in the UK sodium reformulation program.**

| **Food category** | **Sub-category** | **Target (mg/100g)^1^** |
| --- | --- | --- |
| Meat products | Bacon | 1150 (average) |
|  | Ham/other cured meats | 650 (average) |
|  | Sausages (Fresh, chilled, frozen) | 550 |
|  | Cooked sausages and sausage meat products | 680 |
|  | Delicatessen, pork pies and sausage rolls | 450 |
|  | Cornish and meat-based pasties | 400 |
|  | Other meat-based pastry products | 300 |
|  | Cooked uncured meat (Whole muscle) | 270 |
|  | Cooked uncured meat (Reformed whole muscle) | 360 |
|  | Cooked uncured meat (Comminuted or chopped reformed meat) | 540 |
|  | Burgers and grill steaks | 350 |
|  | Canned frankfurters, canned hotdogs and canned burgers | 700 |
|  | Fresh chilled frankfurters | 750 |
| Bread | Bread and rolls | 450 |
|  | Bread and rolls with additions | 450 |
|  | Morning goods – yeast raised | 350 |
|  | Morning goods – powder raised | 500 |
| Breakfast cereals | Breakfast cereals | 400 |
| Cheese | Cheddar and other similar ‘hard pressed’ cheeses | 800 |
|  | Soft white cheese | 270 |
|  | Cottage cheese – plain and flavoured | 210 |
|  | Mozzarella | 540 (average) |
|  | Blue cheese | 800 (average) |
|  | Cheese spreads | 720 |
|  | Other processed cheese | 800 |
| Butter | Salted butters and buttery spreads | 670 |
|  | Lightly salted butter | 450 (average) |
| Fat spreads | Margarines/other spreads | 550 |
| Baked beans | Baked beans in tomato sauce without accompaniments | 225 |
|  | Baked beans and canned pasta with accompaniments | 290 |
| Ready meals and meal centres | Ready meals and meal centres | 380 |
| Soups | Soups (as consumed) | 250 |
| Pizzas | All pizzas (as consumed) | 500 |
| Crisps and snacks | Standard potato crisps | 580 |
|  | Extruded and sheeted snacks | 800 |
|  | Pelleted snacks | 1150 |
|  | Salt and vinegar products | 1000 |
| Cakes, pastries, fruit pies and other pastry-based desserts | Cakes | 280 |
|  | Pastries | 180 |
|  | Sweet pies and other shortcrust or choux pastry-based desserts | 130 |
| Bought sandwiches | Sandwiches with high salt fillings | 600 |
|  | Sandwiches without high salt fillings | 350 |
| Table sauces | Tomato ketchup | 680 |
|  | Brown sauce | 480 |
|  | Salad cream | 630 |
|  | Mayonnaise (not reduced fat/calorie) | 500 |
|  | Mayonnaise (reduced fat/calorie only) | 680 |
|  | Salad dressing | 600 |
| Cook-in and pasta sauces, thick sauces and pastes | Cook in and pasta sauces | 370 |
|  | Pesto and other thick sauces | 650 |
|  | Thick pastes | 1500 |
| Biscuits | Sweet biscuits | 380 |
|  | Savoury biscuits | 700 |
| Pasta | Pasta and noodles, plain and flavoured | 350 |
| Rice | Rice (unflavoured), as consumed | 70 |
|  | Flavoured rice, as consumed | 230 |
| Other cereals | Other cereals | 250 |
| Processed puddings | Dessert mixes, as consumed | 180 |
|  | Cheesecake | 140 |
|  | Sponge-based processed puddings | 250 |
|  | All other processed puddings | 110 |
| Quiche | Quiches | 270 |
| Scotch eggs | Scotch eggs | 310 |
| Canned fish | Canned tuna | 360 (average) |
|  | Canned salmon | 320 (average) |
|  | Other canned fish | 600 |
| Canned vegetables | Canned and bottled vegetables | 50 |
|  | Canned processed, marrowfat and mushy peas | 180 |
| Meat alternatives | Plain meat alternatives | 250 |
|  | Meat-free products | 500 |
|  | Meat-free bacon | 750 |
| Other processed potatoes | Dehydrated instant mashed potato, as consumed | 60 |
|  | Other processed potato products | 275 |
| Beverages | Dried beverages, as consumed | 60 |
| Stocks and gravies | Stocks, as consumed | 380 |
|  | Gravy, as consumed | 450 |

**^1^**The UK maximum salt target is displayed except for targets where no maximum target was set, in which case the average target as indicated by (average), was used as a maximum sodium target

**Text B.** **Comparative risk assessment methods**

*Calculation of potential impact fraction*

In each stratum, we calculated the potential impact fraction (PIF) of reformulation for eight cardiovascular disease subtypes (ischemic heart disease, ischemic stroke, haemorrhagic stroke, aortic aneurysm, endocarditis, hypertensive heart disease, rheumatic heart disease, and other cardiovascular disease [including atrial fibrillation and flutter, cardiomyopathy and myocarditis, peripheral artery disease, and other subtypes]), chronic kidney disease and stomach cancer. The PIF for outcome *o* in age group *a* and sex *s* was calculated as:

$$\mathrm{PIF}_{oas}=\frac{\int_{x=0}^{m} {RR}_{oa}\left( x \right)P_{as}\left( x \right)dx-\int_{x=0}^{m} {RR}_{oa}\left( x \right){P'}_{as}\left( x \right)dx}{\int_{x=0}^{m} {RR}_{oa}\left( x \right)P_{as}\left( x \right)dx}$$

*P_as_(x)* and *P’_as_(x)* are the pre-intervention and post-intervention sodium intake distributions in age group *a* and sex *s*. *RR_oa_(x)* is the relative risk as a function of mmol/d sodium intake (*x*), specific for outcome *o* and age group *a*. For cardiovascular disease subtypes and chronic kidney disease, the *RR_oa_(x)* is defined as:

$${RR}_{oa}(x)=\left\{ \begin{aligned} e^{( {lnRR}_{oa}(y)\cdot\frac{k_{as}}{10} \cdot\frac{x-TMREL}{100})}, &x\geq TMREL \\ 1, &x<TMREL \end{aligned} \right.$$

lnRR_oa_(y) is the increase in the natural logarithm of the relative risk of outcome *o* in age *a* per 10 mm Hg SBP increase (Table E in S1 File), derived from previous meta-analyses [14,16]. TMREL is the theoretical-minimum-risk exposure level, assumed to be uniformly distributed, 87±8.7 mmol/d (i.e., 2.0±0.2 g/d).[17] The k_as_ is the systolic blood pressure effect estimate of sodium reduction in age group *a* and sex *s*, adjusted for hypertension prevalence (measured high blood pressure i.e. SBP≥140 mmHg and/or diastolic BP ≥90 mmHg) and derived from a previous multivariable-adjusted meta-regression of 103 sodium reduction trials [18]. The k_as_ is calculated as

$$k_{as}=P_{as}\left( \alpha+\beta_{1}\left( {age}_{a}-50 \right)+\beta_{2} \right)+\left( 1-P_{as} \right)\left( \alpha+\beta_{1}\left( {age}_{a}-50 \right) \right)$$

Where P_as_ is the hypertension prevalence in age group *a* and sex *s*, α is the blood pressure effect of a 100 mmol/d reduction in sodium intake among non-black, normotensive, 50 year-olds, β_1_ is the additional blood pressure effect of the same sodium reduction for each year above the age of 50 years, age_a_ is the midpoint age of age group (a) and β_2_ is the additional blood pressure effect of a 100 mmol/d sodium reduction among hypertensives compared to normotensives. We used estimates and standard errors (SE) of α, β_1_, and β_2_ determined in a previous meta-regression: α, coefficient (SE) = -3.735 (0.730); β_1_ = -0.105 (0.029); and β_2_ = -1.874 (0.884)[18].

The *RR_a_(x)* for stomach cancer is defined as:

$${RR}_{a}(x)=\left\{ \begin{aligned} e^{( \frac{{lnRR}_{a}(x)\cdot M_{Na}\cdot\left( x-TMREL \right)}{1000})}, &x-TMREL\geq0 \\ 1, &x-TMREL<0 \end{aligned} \right.$$

lnRR_a_(x) is the increase in the natural logarithm of the relative risk of stomach cancer in age *a* per 1 g/d increase of dietary sodium (Table E in S1 File)[19], and M_Na_ is the molar mass of sodium (i.e., 22.99 g/mol).

The PIF for each outcome and stratum was calculated by numerical integration. We assumed no further health benefits for any blood pressure reduction below a threshold of 110 mm Hg.[16]

**Table E.** **Relative risk estimates and corresponding 95% confidence intervals of cardiovascular disease subtypes, chronic kidney disease, and stomach cancer per 10 mmHg increase in systolic blood pressure or 1g/d increase in sodium intake.**

|  |  | **Age interval (y)** | | | | | | | | | | | |
| --- | --- | --- | --- | --- | --- | --- | --- | --- | --- | --- | --- | --- | --- |
| **Risk factor (unit)** | **Disease** | **25-29** | **30-34** | **35-39** | **40-44** | **45-49** | **50-54** | **55-59** | **60-64** | **65-69** | **70-74** | **75-79** | **80+** |
| **Systolic blood pressure (per 10 mmHg)** | Ischemic heart disease^1^ | 1.97  (1.44-2.71) | 1.82  (1.46-2.27) | 1.66  (1.46-1.90) | 1.57  (1.40-1.76) | 1.53  (1.39-1.67) | 1.49  (1.38-1.60) | 1.45  (1.37-1.53) | 1.41 (1.33-1.48) | 1.36 (1.26-1.48) | 1.33 (1.22-1.45) | 1.30 (1.22-1.39) | 1.27 (1.13-1.41) |
|  | Ischemic stroke^1^ | 1.85 (1.39-2.47) | 1.77 (1.43-2.21) | 1.69 (1.40-2.04) | 1.63 (1.35-1.96) | 1.57 (1.36-1.82) | 1.52 (1.36-1.70) | 1.47 (1.34-1.60) | 1.41 (1.30-1.54) | 1.36 (1.21-1.53) | 1.32 (1.17-1.49) | 1.28 (1.18-1.40) | 1.20 (1.11-1.30) |
|  | Haemorrhagic stroke^1^ | 2.13 (1.55-2.93) | 2.05 (1.59-2.64) | 1.97 (1.59-2.43) | 1.87 (1.49-2.36) | 1.78 (1.48-2.13) | 1.68 (1.45-1.94) | 1.58 (1.40-1.78) | 1.48 (1.33-1.64) | 1.38 (1.21-1.58) | 1.32 (1.16-1.51) | 1.31 (1.19-1.44) | 1.28 (1.13-1.45) |
|  | Hypertensive heart disease^2^ | 3.29 (3.00-3.60) | 3.29 (3.00-3.60) | 2.86 (2.67-3.06) | 2.86 (2.67-3.06) | 2.49 (2.37-2.61) | 2.49 (2.37-2.61) | 2.16 (2.09-2.24) | 2.16 (2.09-2.24) | 1.88 (1.82-1.94) | 1.88 (1.82-1.94) | 1.63 (1.56-1.71) | 1.63 (1.56-1.71) |
|  | Aortic aneurysm^1^ | 1.54 (1.26-1.90) | 1.47 (1.29-1.67) | 1.39 (1.30-1.50) | 1.34 (1.23-1.48) | 1.32 (1.23-1.42) | 1.30 (1.23-1.37) | 1.27 (1.22-1.33) | 1.25 (1.19-1.31) | 1.22 (1.16-1.29) | 1.20 (1.14-1.27) | 1.18 (1.13-1.23) | 1.12 (1.07-1.17) |
|  | Rheumatic heart disease^1,3^ | 1.63 (1.17-2.27) | 1.47 (1.17-1.86) | 1.32 (1.14-1.52) | 1.23 (1.09-1.39) | 1.21 (1.10-1.33) | 1.19 (1.11-1.29) | 1.18 (1.10-1.25) | 1.16 (1.09-1.23) | 1.14 (1.05-1.23) | 1.13 (1.05-1.21) | 1.12 (1.06-1.18) | 1.10 (1.04-1.17) |
|  | Endocarditis^1,3^ | 1.76 (1.26-2.43) | 1.61 (1.29-2.00) | 1.46 (1.28-1.66) | 1.37 (1.23-1.52) | 1.34 (1.22-1.46) | 1.31 (1.22-1.40) | 1.28 (1.21-1.34) | 1.25 (1.18-1.32) | 1.22 (1.13-1.31) | 1.19 (1.12-1.28) | 1.18 (1.12-1.23) | 1.13 (1.07-1.19) |
|  | Other CVD^1,4^ | 1.74 (1.34-2.27) | 1.62 (1.38-1.91) | 1.50 (1.41-1.61) | 1.43 (1.35-1.50) | 1.39 (1.34-1.46) | 1.36 (1.32-1.41) | 1.33 (1.30-1.36) | 1.30 (1.27-1.33) | 1.26 (1.23-1.30) | 1.23 (1.20-1.27) | 1.21 (1.18-1.24) | 1.14 (1.09-1.18) |
|  | Chronic kidney disease^5^ | 1.28 (1.18-1.39) | 1.28 (1.18-1.39) | 1.28 (1.18-1.39) | 1.28 (1.18-1.39) | 1.28 (1.18-1.39) | 1.28 (1.18-1.39) | 1.28 (1.18-1.39) | 1.28 (1.18-1.39) | 1.28 (1.18-1.39) | 1.28 (1.18-1.39) | 1.28 (1.18-1.39) | 1.28 (1.18-1.39) |
| **Dietary sodium**  **(per 1 g/d)** | Stomach cancer^6^ | 1.20 (0.99-1.45) | 1.21 (1.01-1.43) | 1.21 (0.99-1.46) | 1.20 (1.00-1.45) | 1.21 (1.00-1.46) | 1.20 (0.99-1.44) | 1.20 (1.01-1.43) | 1.20 (0.99-1.46) | 1.21 (1.01-1.44) | 1.21 (1.00-1.46) | 1.20 (1.00-1.45) | 1.21 (0.99-1.47) |

^1^Risk estimates from Forouzanfar et al., JAMA. 2017;317(2):165-182. doi:10.1001/jama.2016.19043

^2^Risk estimates from Singh et al. PLOS ONE. 2013; 8(7): e65174.

^3^Rheumatic heart disease, endocarditis, and myocarditis will included in the modelling of CVD mortality but not incident CVD or CVD-attributed DALYs.

^4^Other CVD: Aortic aneurysm, atrial fibrillation and flutter, cardiomyopathy and myocarditis, endocarditis, hypertensive heart disease, miscellaneous cardiovascular disease, peripheral artery disease and rheumatic heart disease.

^5^Risk estimates from The Global Burden of Metabolic Risk Factors for Chronic Diseases Collaboration. Lancet Diabetes Endocrinol. 2014; 2(8): 634-647.

^6^Risk estimates from GBD 2017 Diet Collaborators. Lancet. 2019; 393: 1958–72.

**Table F.** **Estimated number of deaths (95% uncertainty intervals) in Australia 2017 per disease^1^, stratified by age and sex.**

|  | **Age interval (y)** | | | | | | | | | | | |
| --- | --- | --- | --- | --- | --- | --- | --- | --- | --- | --- | --- | --- |
| **Disease outcome** | **25-29** | **30-34** | **35-39** | **40-44** | **45-49** | **50-54** | **55-59** | **60-64** | **65-69** | **70-74** | **75-79** | **80+** |
| **Men** |  |  |  |  |  |  |  |  |  |  |  |  |
| Aortic aneurysm | 1  (1-1) | 2  (2-1) | 3  (4-2) | 5  (6-3) | 9  (12-7) | 14  (18-10) | 22  (29-17) | 42  (54-32) | 68  (89-52) | 101  (131-77) | 130  (166-101) | 395  (465-335) |
| Atrial fibrillation and flutter | 0  (0-0) | 0  (0-0) | 0  (0-0) | 1  (2-1) | 2  (3-2) | 5  (6-4) | 12  (15-9) | 23  (29-17) | 46  (57-33) | 92  (114-62) | 140  (174-96) | 882  (1,025-610) |
| Cardiomyopathy and myocarditis | 10  (14-7) | 13  (19-9) | 19  (28-13) | 29  (42-21) | 38  (59-27) | 43  (77-30) | 65  (104-47) | 83  (137-59) | 94  (153-67) | 106  (152-78) | 96  (136-72) | 276  (343-227) |
| Endocarditis | 2  (2-1) | 3  (4-2) | 3  (5-2) | 5  (7-3) | 6  (8-4) | 8  (11-5) | 11  (15-8) | 12  (16-9) | 21  (29-15) | 24  (32-17) | 29  (38-21) | 115  (136-94) |
| Hypertensive heart disease | 0  (1-0) | 1  (1-0) | 2  (3-1) | 4  (6-2) | 7  (10-4) | 11  (15-7) | 17  (23-11) | 21  (29-14) | 31  (43-20) | 37  (54-26) | 48  (68-35) | 249  (303-189) |
| Intracerebral hemorrhage^2^ | 1  (2-1) | 3  (5-2) | 6  (9-4) | 12  (18-8) | 22  (30-15) | 35  (46-26) | 60  (77-45) | 86  (110-65) | 148  (191-113) | 221  (283-169) | 331  (414-256) | 1,201  (1,398-1,024) |
| Subarachnoid hemorrhage^2^ | 3  (4-2) | 5  (7-3) | 9  (12-6) | 14  (19-10) | 20  (28-14) | 26  (36-19) | 32  (44-23) | 33  (44-24) | 43  (57-31) | 47  (64-35) | 56  (73-42) | 210  (251-146) |
| Ischemic heart disease | 10  (14-7) | 30  (38-22) | 72  (90-56) | 136  (169-107) | 266  (325-215) | 419  (509-341) | 605  (726-503) | 811  (960-678) | 1,145  (1,355-961) | 1,472  (1,740-1,247) | 1,790  (2,105-1,510) | 8,471  (9,435-7,662) |
| Ischemic stroke | 0  (1-0) | 1  (1-0) | 2  (2-1) | 3  (4-2) | 7  (10-5) | 12  (16-8) | 25  (34-18) | 52  (70-39) | 90  (118-68) | 182  (238-137) | 298  (376-228) | 2,197  (2,499-1,924) |
| Other CVD^3^ | 2  (3-1) | 3  (5-2) | 5  (7-4) | 9  (12-6) | 14  (20-10) | 17  (23-13) | 28  (37-21) | 38  (49-29) | 49  (63-37) | 57  (73-42) | 62  (80-47) | 186  (218-158) |
| Peripheral artery disease | 0  (0-0) | 0  (0-0) | 0  (0-0) | 1  (1-0) | 1  (1-0) | 3  (6-1) | 6  (12-2) | 16  (33-7) | 31  (68-13) | 56  (114-23) | 74  (152-31) | 353  (679-180) |
| Rheumatic heart disease | 1  (2-1) | 2  (2-1) | 2  (3-1) | 2  (3-1) | 3  (5-2) | 5  (7-3) | 7  (9-5) | 8  (11-6) | 12  (16-9) | 19  (24-14) | 23  (29-18) | 84  (99-72) |
| Chronic kidney disease | 2  (3-2) | 3  (4-2) | 5  (7-4) | 10  (14-7) | 18  (24-14) | 28  (36-21) | 45  (57-35) | 70  (89-55) | 112  (142-87) | 191  (240-152) | 280  (354-222) | 1,450  (1,644-1,264) |
| Stomach cancer | 1  (1-1) | 3  (4-2) | 6  (8-4) | 14  (18-10) | 24  (32-19) | 40  (50-31) | 70  (87-55) | 97  (119-78) | 134  (165-108) | 166  (203-135) | 174  (214-142) | 399  (458-347) |
| **Women** |  |  |  |  |  |  |  |  |  |  |  |  |
| Aortic aneurysm | 0  (1-0) | 1  (1-0) | 1  (2-1) | 2  (2-1) | 3  (4-2) | 4  (6-3) | 8  (12-6) | 16  (20-12) | 29  (38-22) | 51  (67-37) | 76  (98-58) | 335  (387-288) |
| Atrial fibrillation and flutter | 0  (0-0) | 0  (0-0) | 0  (0-0) | 0  (1-0) | 1  (2-1) | 3  (3-2) | 7  (9-5) | 17  (22-13) | 36  (46-29) | 89  (111-71) | 162  (195-132) | 1,850  (2,076-1,635) |
| Cardiomyopathy and myocarditis | 3  (4-2) | 4  (6-3) | 6  (8-4) | 7  (10-5) | 11  (15-8) | 13  (17-9) | 15  (21-10) | 22  (28-16) | 27  (36-20) | 34  (45-25) | 43  (57-31) | 248  (292-209) |
| Endocarditis | 1  (2-1) | 2  (2-1) | 1  (3-1) | 2  (4-1) | 3  (5-2) | 3  (5-1) | 6  (9-3) | 7  (11-3) | 10  (15-5) | 18  (27-8) | 23  (33-10) | 139  (183-69) |
| Hypertensive heart disease | 0  (0-0) | 0  (1-0) | 1  (1-1) | 2  (3-1) | 3  (5-2) | 5  (8-3) | 7  (13-4) | 11  (21-7) | 16  (32-10) | 28  (49-18) | 48  (81-32) | 650  (798-393) |
| Intracerebral hemorrhage^2^ | 1  (2-1) | 3  (4-2) | 4  (6-3) | 7  (10-5) | 14  (18-10) | 23  (31-17) | 33  (44-24) | 50  (66-37) | 80  (104-60) | 146  (187-109) | 260  (331-201) | 1,786  (2,074-1,536) |
| Subarachnoid hemorrhage^2^ | 2  (3-2) | 5  (7-4) | 10  (13-7) | 16  (21-12) | 26  (34-19) | 31  (40-23) | 36  (49-27) | 42  (56-31) | 50  (69-37) | 52  (72-37) | 82  (108-61) | 352  (415-294) |
| Ischemic heart disease | 3  (4-2) | 8  (10-6) | 16  (21-12) | 30  (38-23) | 61  (77-46) | 84  (106-67) | 139  (172-111) | 217  (270-171) | 349  (429-282) | 571  (699-459) | 928  (1,115-756) | 10,057  (11,119-8,996) |
| Ischemic stroke | 0  (1-0) | 1  (1-1) | 1  (2-1) | 2  (3-1) | 4  (5-3) | 5  (7-4) | 10  (13-7) | 24  (32-18) | 46  (61-33) | 104  (139-76) | 210  (271-162) | 3,485  (3,921-3,065) |
| Other CVD^3^ | 3  (4-2) | 4  (5-3) | 6  (8-4) | 9  (12-6) | 13  (17-9) | 16  (22-12) | 19  (26-14) | 27  (35-20) | 37  (48-28) | 48  (62-36) | 52  (68-40) | 231  (268-196) |
| Peripheral artery disease | 0  (0-0) | 0  (0-0) | 0  (0-0) | 0  (0-0) | 0  (1-0) | 3  (7-1) | 4  (11-1) | 9  (23-3) | 19  (54-6) | 34  (95-10) | 57  (156-19) | 621  (1,615-256) |
| Rheumatic heart disease | 1  (2-1) | 2  (3-1) | 3  (4-2) | 4  (5-2) | 5  (7-3) | 6  (9-4) | 9  (12-6) | 13  (18-10) | 17  (23-13) | 29  (37-22) | 38  (49-28) | 212  (244-184) |
| Chronic kidney disease | 1  (2-1) | 3  (4-2) | 5  (6-3) | 7  (9-5) | 12  (16-9) | 21  (28-16) | 35  (46-26) | 51  (64-38) | 80  (103-61) | 140  (173-108) | 209  (261-164) | 1,671  (1,913-1,462) |
| Stomach cancer | 1  (2-1) | 3  (4-2) | 7  (10-5) | 12  (15-9) | 16  (21-12) | 24  (30-19) | 33  (42-26) | 48  (59-38) | 61  (76-49) | 83  (105-66) | 94  (118-74) | 368  (420-320) |

^1^Data retrieved from GBD Results Tool (<http://ghdx.healthdata.org/gbd-results-tool>). Access date: July 6^th^, 2020.

^2^Intracerebral haemorrhage and Subarachnoid haemorrhage were aggregated to estimate haemorrhagic stroke.
^3^Other CVD: Aortic aneurysm, atrial fibrillation and flutter, cardiomyopathy and myocarditis, endocarditis, hypertensive heart disease, miscellaneous cardiovascular disease, peripheral artery disease and rheumatic heart disease.

**Table G. Estimated number of incidences (95% uncertainty intervals) in Australia 2017 per disease^1^, stratified by age and sex.**

|  | **Age interval (y)** | | | | | | | | | | | |  |
| --- | --- | --- | --- | --- | --- | --- | --- | --- | --- | --- | --- | --- | --- |
| **Disease outcome** | **25-29** | **30-34** | **35-39** | **40-44** | **45-49** | **50-54** | **55-59** | **60-64** | **65-69** | **70-74** | **75-79** | **80+** | |
| **Men** |  |  |  |  |  |  |  |  |  |  |  |  | |
| Atrial fibrillation and flutter | 0  (0-0) | 57  (76-40) | 94  (140-55) | 206  (272-150) | 410  (575-265) | 747  (965-559) | 1,250  (1,811-788) | 1,956  (2,476-1,464) | 2,835  (3,949-1,855) | 2,746  (3,598-2,032) | 1,936  (2,647-1,317) | 1,598  (2,110-1,098) | |
| Cardiomyopathy and myocarditis | 216  (291-148) | 217  (292-150) | 200  (276-138) | 206  (286-139) | 246  (341-169) | 281  (379-205) | 343  (478-245) | 391  (527-295) | 451  (605-307) | 469  (611-352) | 410  (550-292) | 633  (792-497) | |
| Endocarditis | 25  (37-16) | 30  (42-20) | 32  (47-20) | 39  (53-26) | 53  (80-33) | 72  (98-52) | 103  (145-70) | 141  (182-106) | 189  (246-140) | 226  (279-179) | 220  (279-168) | 426  (492-362) | |
| Intracerebral hemorrhage^2^ | 17  (32-5) | 29  (46-15) | 45  (69-25) | 64  (87-44) | 93  (129-64) | 130  (168-99) | 187  (239-140) | 238  (289-195) | 298  (391-216) | 361  (442-293) | 366  (460-287) | 1,163  (1,318-1,016) | |
| Subarachnoid hemorrhage^2^ | 24  (35-16) | 45  (58-34) | 71  (97-48) | 102  (132-78) | 148  (194-109) | 164  (206-122) | 171  (229-122) | 168  (225-122) | 171  (222-126) | 147  (186-113) | 102  (132-78) | 148  (181-123) | |
| Ischemic heart disease | 46  (94-11) | 187  (262-119) | 422  (624-255) | 986  (1,282-738) | 1,969  (2,706-1,359) | 2,977  (3,707-2,298) | 4,250  (5,747-2,908) | 5,076  (6,504-3,919) | 5,850  (8,033-4,265) | 5,874  (7,695-4,597) | 4,884  (6,638-3,649) | 10,580  (12,435-9,068) | |
| Ischemic stroke | 44  (84-14) | 80  (125-45) | 125  (200-66) | 212  (293-146) | 357  (522-229) | 553  (735-405) | 839  (1,209-574) | 1,123  (1,467-866) | 1,454  (1,971-1,043) | 1,568  (1,991-1,196) | 1,366  (1,764-1,029) | 3,362  (3,906-2,864) | |
| Peripheral artery disease | 0  (0-0) | 0  (0-0) | 0  (0-0) | 595  (740-472) | 803  (1,204-523) | 1,261  (1,644-947) | 2,038  (2,841-1,344) | 2,563  (3,311-1,936) | 3,015  (4,143-2,095) | 2,594  (3,484-1,843) | 1,626  (2,292-1,131) | 2,014  (2,690-1,509) | |
| Rheumatic heart disease | 1  (4-0) | 3  (5-1) | 4  (8-2) | 6  (9-4) | 9  (14-4) | 12  (18-7) | 17  (25-11) | 20  (27-15) | 24  (32-16) | 27  (33-21) | 26  (32-21) | 87  (99-78) | |
| Chronic kidney disease | 122  (217-37) | 191  (340-77) | 342  (583-160) | 612  (959-358) | 1,014  (1,503-646) | 1,468  (2,084-920) | 2,417  (3,540-1,509) | 3,469  (4,847-2,312) | 5,005  (7,002-3,450) | 6,464  (9,052-4,568) | 5,509  (7,623-3,666) | 5,869  (7,739-4,354) | |
| Stomach cancer | 4  (5-3) | 11  (15-8) | 21  (29-15) | 44  (61-28) | 67  (93-43) | 102  (135-70) | 167  (225-110) | 235  (299-161) | 309  (391-224) | 349  (442-253) | 333  (421-245) | 629  (743-483) | |
| **Women** |  |  |  |  |  |  |  |  |  |  |  |  | |
| Atrial fibrillation and flutter | 0  (0-0) | 20  (28-12) | 27  (45-12) | 72  (99-49) | 162  (237-103) | 368  (487-270) | 711  (1,029-442) | 1,342  (1,686-1,008) | 2,162  (2,930-1,390) | 2,301  (2,939-1,733) | 1,893  (2,652-1,251) | 2,599  (3,391-1,944) | |
| Cardiomyopathy and myocarditis | 133  (181-90) | 154  (204-107) | 170  (234-117) | 202  (270-145) | 260  (363-175) | 308  (406-227) | 391  (561-275) | 462  (620-349) | 543  (745-377) | 570  (739-428) | 523  (712-372) | 1,137  (1,450-901) | |
| Endocarditis | 26  (38-17) | 31  (43-20) | 29  (43-19) | 32  (46-21) | 41  (62-26) | 48  (68-33) | 62  (90-40) | 81  (106-56) | 106  (147-70) | 130  (167-100) | 139  (179-99) | 421  (498-351) | |
| Intracerebral hemorrhage^2^ | 22  (39-9) | 32  (52-16) | 40  (65-20) | 55  (80-35) | 78  (115-49) | 106  (142-76) | 150  (203-103) | 183  (231-140) | 214  (288-151) | 288  (361-235) | 343  (436-265) | 1,779  (2,040-1,563) | |
| Subarachnoid hemorrhage^2^ | 31  (44-20) | 65  (83-49) | 109  (149-77) | 165  (214-128) | 245  (327-181) | 274  (345-211) | 298  (394-219) | 303  (384-225) | 315  (407-228) | 279  (349-216) | 210  (272-160) | 376  (455-307) | |
| Ischemic heart disease | 19  (48-3) | 42  (76-18) | 78  (143-30) | 192  (263-131) | 402  (579-261) | 648  (842-472) | 997  (1,438-628) | 1,416  (1,833-1,062) | 1,919  (2,692-1,310) | 2,248  (2,874-1,730) | 2,237  (3,194-1,557) | 9,441  (10,946-8,149) | |
| Ischemic stroke | 58  (104-24) | 91  (141-51) | 119  (193-64) | 176  (252-114) | 276  (415-172) | 381  (525-274) | 539  (797-365) | 822  (1,080-625) | 1,213  (1,670-815) | 1,368  (1,760-1,028) | 1,261  (1,660-919) | 5,026  (5,805-4,275) | |
| Peripheral artery disease | 0  (0-0) | 0  (0-0) | 0  (0-0) | 1,188  (1,452-942) | 1,077  (1,566-723) | 1,237  (1,674-875) | 1,831  (2,642-1,173) | 2,524  (3,294-1,879) | 3,325  (4,579-2,266) | 3,115  (4,132-2,224) | 2,255  (3,152-1,540) | 3,620  (4,693-2,709) | |
| Rheumatic heart disease | 2  (5-0) | 4  (7-2) | 6  (10-2) | 8  (12-5) | 12  (19-6) | 16  (23-11) | 23  (33-14) | 28  (36-21) | 33  (43-23) | 39  (47-31) | 42  (53-33) | 220  (245-197) | |
| Chronic kidney disease | 204  (363-80) | 338  (590-171) | 588  (970-327) | 1,061  (1,661-622) | 1,832  (2,596-1,197) | 2,637  (3,712-1,698) | 4,218  (6,077-2,576) | 5,722  (8,112-3,619) | 7,647  (10,666-5,064) | 9,150  (13,157-6,486) | 7,875  (11,004-5,286) | 10,125  (12,949-7,702) | |
| Stomach cancer | 5  (7-3) | 11  (15-8) | 24  (34-16) | 33  (48-20) | 42  (60-26) | 50  (75-33) | 72  (102-47) | 103  (144-67) | 138  (180-97) | 167  (224-116) | 175  (227-122) | 578  (682-434) | |

^1^Data retrieved from GBD Results Tool (<http://ghdx.healthdata.org/gbd-results-tool>). Access date: July 6^th^, 2020.

^2^Intracerebral haemorrhage and Subarachnoid haemorrhage were aggregated to estimate haemorrhagic stroke.

**Table H.** **Estimated number of DALYs (95% uncertainty intervals) in Australia 2017 per disease^1^, age, and sex.**

|  | **Age interval (y)** | | | | | | | | | | | |
| --- | --- | --- | --- | --- | --- | --- | --- | --- | --- | --- | --- | --- |
| **Disease outcome** | **25-29** | **30-34** | **35-39** | **40-44** | **45-49** | **50-54** | **55-59** | **60-64** | **65-69** | **70-74** | **75-79** | **80+** |
| **Men** |  |  |  |  |  |  |  |  |  |  |  |  |
| Aortic aneurysm | 59  (86-39) | 89  (124-60) | 140  (199-96) | 210  (295-150) | 375  (512-273) | 496  (663-365) | 696  (904-525) | 1,119  (1,441-853) | 1,524  (1,995-1,155) | 1,832  (2,374-1,387) | 1,825  (2,329-1,412) | 3,050  (3,663-2,550) |
| Atrial fibrillation and flutter | 0  (0-0) | 85  (154-33) | 118  (205-55) | 211  (325-127) | 383  (566-240) | 662  (944-455) | 1,230  (1,685-875) | 1,953  (2,650-1,428) | 3,123  (4,124-2,295) | 4,308  (5,582-3,291) | 4,440  (5,606-3,485) | 10,151  (12,070-7,998) |
| Cardiomyopathy and myocarditis | 2,449  (3,011-2,014) | 648  (893-463) | 800  (1,109-577) | 1,040  (1,491-727) | 1,430  (1,965-1,047) | 1,709  (2,555-1,236) | 1,783  (2,997-1,286) | 2,320  (3,536-1,724) | 2,567  (4,035-1,888) | 2,566  (3,890-1,906) | 2,321  (3,196-1,757) | 1,615  (2,201-1,248) |
| Endocarditis | 104  (152-64) | 151  (230-96) | 155  (234-92) | 220  (320-144) | 245  (352-166) | 290  (406-203) | 350  (469-258) | 341  (457-248) | 490  (664-366) | 458  (603-339) | 437  (568-323) | 939  (1,119-769) |
| Hypertensive heart disease | 21  (33-9) | 53  (77-32) | 96  (142-58) | 181  (265-122) | 309  (440-196) | 427  (597-289) | 579  (770-413) | 642  (885-444) | 833  (1,114-567) | 858  (1,192-635) | 843  (1,116-627) | 2,078  (2,541-1,692) |
| Intracerebral hemorrhage^2^ | 119  (167-80) | 238  (332-167) | 363  (506-253) | 634  (882-438) | 1,002  (1,347-715) | 1,392  (1,816-1,069) | 2,061  (2,605-1,586) | 2,525  (3,151-1,929) | 3,506  (4,500-2,727) | 4,164  (5,277-3,262) | 4,759  (5,914-3,689) | 8,952  (10,512-7,583) |
| Subarachnoid hemorrhage^2^ | 194  (268-139) | 319  (427-232) | 508  (689-376) | 743  (968-559) | 998  (1,322-735) | 1,165  (1,525-876) | 1,252  (1,649-958) | 1,091  (1,395-823) | 1,111  (1,449-850) | 966  (1,280-740) | 855  (1,097-652) | 1,665  (2,001-1,226) |
| Ischemic heart disease | 645  (843-474) | 1,694  (2,173-1,282) | 3,710  (4,650-2,926) | 6,375  (7,855-5,083) | 11,182  (13,672-9,143) | 15,627  (18,900-12,804) | 19,750  (23,564-16,527) | 22,786  (26,891-19,241) | 27,088  (31,774-22,913) | 28,323  (33,190-24,186) | 26,668  (31,119-22,871) | 63,184  (70,451-56,976) |
| Ischemic stroke | 124  (185-80) | 178  (254-123) | 256  (391-153) | 393  (567-258) | 713  (952-504) | 1,041  (1,366-774) | 1,771  (2,246-1,370) | 2,765  (3,391-2,191) | 3,888  (4,747-3,079) | 5,725  (7,067-4,588) | 6,862  (8,232-5,683) | 21,336  (24,040-18,658) |
| Other CVD^3^ | 203  (290-136) | 251  (348-176) | 392  (546-276) | 688  (928-507) | 942  (1,267-695) | 1,048  (1,409-770) | 1,583  (2,057-1,174) | 2,073  (2,718-1,564) | 2,317  (3,065-1,731) | 2,346  (3,094-1,720) | 2,005  (2,706-1,478) | 2,465  (3,078-1,981) |
| Peripheral artery disease | 0  (0-0) | 0  (0-0) | 0  (0-0) | 27  (57-6) | 29  (58-13) | 116  (230-54) | 232  (423-118) | 523  (986-265) | 836  (1,679-409) | 1,154  (2,196-570) | 1,140  (2,215-533) | 2,677  (4,986-1,430) |
| Rheumatic heart disease | 87  (121-58) | 93  (131-65) | 105  (151-70) | 90  (127-60) | 134  (194-93) | 177  (248-124) | 217  (293-159) | 223  (296-166) | 287  (370-220) | 344  (440-268) | 329  (411-261) | 657  (783-558) |
| Chronic kidney disease | 347  (474-251) | 481  (669-337) | 652  (872-479) | 950  (1,206-722) | 1,422  (1,864-1,097) | 1,730  (2,255-1,337) | 2,289  (2,811-1,807) | 2,857  (3,452-2,324) | 3,663  (4,495-2,946) | 4,736  (5,781-3,902) | 4,933  (5,973-4,088) | 12,184  (13,869-10,606) |
| Stomach cancer | 63  (89-44) | 177  (238-124) | 305  (416-219) | 646  (836-488) | 1,028  (1,318-802) | 1,474  (1,848-1,156) | 2,259  (2,830-1,796) | 2,701  (3,296-2,150) | 3,108  (3,826-2,482) | 3,106  (3,832-2,523) | 2,542  (3,126-2,058) | 3,393  (3,918-2,920) |
| **Women** |  |  |  |  |  |  |  |  |  |  |  |  |
| Aortic aneurysm | 20  (30-13) | 40  (58-25) | 53  (77-34) | 70  (101-48) | 122  (165-84) | 154  (213-108) | 261  (369-179) | 417  (550-309) | 648  (853-482) | 911  (1,213-675) | 1,056  (1,369-806) | 2,423  (2,858-2,039) |
| Atrial fibrillation and flutter | 0  (0-0) | 49  (99-14) | 55  (117-18) | 88  (148-42) | 174  (271-101) | 301  (429-200) | 636  (894-463) | 1,197  (1,592-897) | 2,117  (2,752-1,588) | 3,440  (4,314-2,687) | 4,212  (5,135-3,387) | 15,971  (18,042-13,929) |
| Cardiomyopathy and myocarditis | 2,141  (2,512-1,789) | 210  (280-149) | 293  (405-204) | 353  (466-247) | 417  (559-306) | 581  (764-425) | 607  (806-449) | 652  (873-482) | 786  (1,010-604) | 873  (1,130-675) | 850  (1,099-643) | 821  (1,041-631) |
| Endocarditis | 72  (110-40) | 87  (130-48) | 77  (128-37) | 108  (175-61) | 127  (201-72) | 120  (182-55) | 186  (280-98) | 217  (322-108) | 239  (352-120) | 348  (513-178) | 351  (498-172) | 1,033  (1,343-549) |
| Hypertensive heart disease | 9  (13-6) | 30  (44-21) | 55  (80-36) | 96  (154-62) | 144  (219-94) | 210  (335-138) | 282  (468-194) | 388  (666-283) | 523  (883-369) | 728  (1,137-529) | 917  (1,383-667) | 4,746  (5,901-3,353) |
| Intracerebral hemorrhage^2^ | 132  (167-99) | 231  (314-165) | 308  (411-217) | 446  (571-332) | 717  (918-542) | 1,012  (1,305-775) | 1,288  (1,638-968) | 1,611  (2,046-1,254) | 2,077  (2,655-1,589) | 2,898  (3,673-2,238) | 3,824  (4,830-2,980) | 11,947  (13,992-10,201) |
| Subarachnoid hemorrhage^2^ | 198  (258-149) | 372  (481-280) | 610  (797-457) | 924  (1,166-700) | 1,363  (1,708-1,046) | 1,537  (1,953-1,201) | 1,712  (2,158-1,338) | 1,740  (2,179-1,382) | 1,664  (2,137-1,275) | 1,325  (1,714-1,012) | 1,361  (1,752-1,065) | 2,653  (3,117-2,222) |
| Ischemic heart disease | 184  (247-132) | 472  (623-347) | 880  (1,149-661) | 1,490  (1,878-1,174) | 2,740  (3,413-2,163) | 3,378  (4,164-2,731) | 4,880  (5,908-3,955) | 6,491  (7,893-5,211) | 8,769  (10,522-7,252) | 11,539  (13,924-9,476) | 14,266  (16,882-11,849) | 64,673  (71,944-57,591) |
| Ischemic stroke | 159  (234-101) | 235  (372-129) | 309  (462-179) | 425  (612-276) | 650  (915-435) | 858  (1,178-594) | 1,316  (1,756-943) | 2,011  (2,547-1,538) | 2,882  (3,565-2,232) | 4,261  (5,187-3,437) | 5,651  (6,796-4,660) | 29,425  (32,875-25,937) |
| Other CVD^3^ | 348  (490-240) | 410  (586-287) | 595  (812-425) | 1,162  (1,697-782) | 1,263  (1,755-911) | 1,418  (1,994-1,031) | 1,565  (2,157-1,139) | 1,946  (2,574-1,409) | 2,085  (2,832-1,506) | 2,011  (2,702-1,511) | 1,796  (2,382-1,323) | 2,781  (3,416-2,274) |
| Peripheral artery disease | 0  (0-0) | 0  (0-0) | 0  (0-0) | 5  (12-3) | 22  (55-8) | 110  (277-42) | 177  (389-80) | 327  (719-143) | 559  (1,336-233) | 775  (1,863-323) | 924  (2,302-387) | 4,103  (10,284-1,790) |
| Rheumatic heart disease | 92  (126-64) | 120  (163-84) | 146  (208-101) | 172  (250-116) | 209  (293-143) | 231  (326-158) | 279  (378-197) | 363  (493-270) | 396  (523-293) | 528  (686-403) | 543  (695-403) | 1,438  (1,666-1,231) |
| Chronic kidney disease | 432  (602-298) | 656  (948-453) | 881  (1,230-629) | 997  (1,361-708) | 1,392  (1,861-1,021) | 1,619  (2,148-1,224) | 2,087  (2,653-1,620) | 2,460  (3,103-1,910) | 3,330  (4,584-2,468) | 3,987  (5,069-3,162) | 4,349  (5,419-3,509) | 13,791  (15,994-11,975) |
| Stomach cancer | 78  (108-55) | 184  (249-129) | 354  (491-246) | 539  (678-420) | 667  (856-499) | 888  (1,120-702) | 1,073  (1,342-839) | 1,313  (1,632-1,054) | 1,421  (1,748-1,136) | 1,558  (1,962-1,231) | 1,371  (1,724-1,077) | 2,837  (3,280-2,426) |

^1^Data retrieved from GBD Results Tool (<http://ghdx.healthdata.org/gbd-results-tool>). Access date: July 6^th^, 2020.

^2^Intracerebral haemorrhage and Subarachnoid haemorrhage were aggregated to estimate haemorrhagic stroke.
^3^Other CVD: Aortic aneurysm, atrial fibrillation and flutter, cardiomyopathy and myocarditis, endocarditis, hypertensive heart disease, miscellaneous cardiovascular disease, peripheral artery disease and rheumatic heart disease.

| **Assumption/input in primary model** | **Assumption/input in sensitivity analysis** |
| --- | --- |
| TMREL^1^=2.0±0.2 g sodium /d | TMREL^1^=1.0±0.2 g sodium /d  TMREL^1^=3.0±0.6 g sodium /d |
| Estimated current sodium intake is based on a systematic review of 24-hour urine sodium excretion in Australia [6] | Assume sodium intake is 10% lower than the estimated current 24-hour urine sodium excretion. |
| 85% of daily sodium intake comes from packaged, processed and foods consumed out of home.[7-9] | 75% of daily sodium intake comes from packaged, processed, and foods consumed out of home. |

**Table I.** **Model inputs and assumptions in sensitivity analyses**

^1^TMREL is the theoretical-minimum-risk exposure level

**Table J. Estimated sodium reductions from full compliance across each reformulation scenario, stratified by age and sex.**

|  | **Australia targets** | | **UK targets** | | **Optimistic reformulation^1^** | |
| --- | --- | --- | --- | --- | --- | --- |
| **Age, y** | **Mean sodium reduction (mg/day)** | **% reduction^2^** | **Mean sodium reduction (mg/day)** | **% reduction^2^** | **Mean sodium reduction (mg/day)** | **% reduction^2^** |
| **Men** |  |  |  |  |  |  |
| 25 to 29 | 116.7 | 3.1 | 213.2 | 5.7 | 349.7 | 9.4 |
| 30 to 34 | 135.5 | 3.6 | 259.3 | 7.0 | 368.7 | 9.9 |
| 35 to 39 | 154.7 | 3.9 | 278.1 | 7.0 | 382.9 | 9.6 |
| 40 to 44 | 125.2 | 3.5 | 249.8 | 7.0 | 370.7 | 10.4 |
| 45 to 49 | 117.9 | 3.5 | 252.4 | 7.5 | 376.9 | 11.2 |
| 50 to 54 | 104.0 | 3.0 | 241.7 | 6.9 | 320.6 | 9.1 |
| 55 to 59 | 127.7 | 4.2 | 314.5 | 10.3 | 411.1 | 13.5 |
| 60 to 64 | 132.5 | 4.5 | 270.9 | 9.2 | 354.8 | 12.0 |
| 65 to 69 | 116.2 | 4.0 | 260.9 | 8.9 | 364.6 | 12.4 |
| 70 to 74 | 124.4 | 4.2 | 296.4 | 10.1 | 409.6 | 13.9 |
| 75 to 79 | 129.7 | 4.8 | 254.6 | 9.3 | 361.8 | 13.2 |
| 80+ | 112.7 | 4.3 | 264.6 | 10.1 | 357.5 | 13.6 |
| **Women** |  |  |  |  |  |  |
| 25 to 29 | 94.0 | 3.5 | 178.9 | 6.6 | 276.8 | 10.3 |
| 30 to 34 | 102.0 | 3.9 | 187.6 | 7.2 | 306.9 | 11.8 |
| 35 to 39 | 89.1 | 3.3 | 182.4 | 6.7 | 280.2 | 10.3 |
| 40 to 44 | 86.7 | 3.4 | 170.7 | 6.7 | 279.1 | 11.0 |
| 45 to 49 | 100.1 | 4.0 | 177.2 | 7.1 | 270.8 | 10.8 |
| 50 to 54 | 76.2 | 3.1 | 182.2 | 7.5 | 278.7 | 11.5 |
| 55 to 59 | 73.5 | 3.2 | 152.2 | 6.6 | 229.7 | 9.9 |
| 60 to 64 | 93.2 | 3.9 | 201.9 | 8.5 | 304.5 | 12.8 |
| 65 to 69 | 88.7 | 4.0 | 171.5 | 7.6 | 280.5 | 12.5 |
| 70 to 74 | 83.1 | 4.0 | 198.4 | 9.6 | 268.5 | 13.0 |
| 75 to 79 | 71.5 | 3.5 | 185.9 | 9.0 | 256.0 | 12.4 |
| 80+ | 89.9 | 4.0 | 232.7 | 10.5 | 305.5 | 13.7 |

^1^Optimistic reformulation = Reduction to the current 25^th^ sales-weighted percentile sodium content for each of the 80 food categories that make up the UK targets. ^2^ Percent reduction of total sodium from non-discretionary sources.

**Fig A.** **Estimated effects of full compliance to the Australian government’s sodium targets on cardiovascular disease, chronic kidney disease and stomach cancer mortality by age (<70 years, ≤70 years) and sex (men, women).** Values are central estimates (median) of n=1,000 simulations and error bars indicate 95% uncertainty intervals.

**Table K.** **Estimated intervention effects on cardiovascular disease, chronic kidney disease, and stomach cancer in the adult Australian population by the Australian government’s sodium reformulation targets compared with the UK sodium targets and optimistic reformulation scenario.**

|  | **Additional averted no. of events**  **Median (95% UI)** | | |
| --- | --- | --- | --- |
| **Metric and disease** | **UK targets vs Australia’s targets** | **Optimistic reformulation^1^ vs Australia’s targets** | |
| **Deaths/year** |  |  | |
| Total | 660 (207, 1227) | 1065 (511, 1856) | |
| Cardiovascular disease | 541 (155, 1063) | 884 (382, 1599) | |
| Stroke | 132 (32, 255) | 209 (80, 394) | |
| Ischaemic heart disease | 321 (70, 674) | 519 (208, 990) | |
| Other^2^ | 89 (25, 170) | 146 (62, 256) | |
| Chronic kidney disease | 50 (10, 107) | 80 (29, 157) | |
| Stomach cancer | 63 (1, 135) | 99 (27, 192) | |
| **Incidences^3/^year** |  |  | |
| Total | 2341 (785, 4046) | 3929 (1907, 6098) | |
| Cardiovascular disease | 1539 (469, 2656) | 2535 (1170, 4082) | |
| Stroke | 340 (95, 600) | 563 (251, 921) | |
| Ischaemic heart disease | 733 (207, 1336) | 1194 (536, 2041) | |
| Other | 453 (139, 770) | 770 (361, 1218) | |
| Chronic kidney disease | 687 (179, 1295) | 1171 (514, 1935) | |
| Stomach cancer | 118 (13, 264) | 195 (69, 363) | |
| **Disability adjusted life years/year** | | |  |
| Total | 8748 (3109, 15552) | 14435 (7538, 22143) | |
| Cardiovascular disease | 7042 (2054, 12759) | 11542 (5338, 18468) | |
| Stroke | 1809 (513, 3453) | 3057 (1355, 5032) | |
| Ischaemic heart disease | 3973 (1095, 7600) | 6386 (2909, 10636) | |
| Other | 1228 (348, 2261) | 2004 (908, 3193) | |
| Chronic kidney disease | 680 (176, 1258) | 1087 (507, 1822) | |
| Stomach cancer | 1088 (245, 2142) | 1804 (689, 3116) | |

^1^Optimistic reformulation = Reduction to the current 25^th^ sales-weighted percentile sodium content for each of the 80 food categories that make up the UK targets. ^2^Other CVD: Aortic aneurysm, atrial fibrillation and flutter, cardiomyopathy and myocarditis, endocarditis, hypertensive heart disease, miscellaneous cardiovascular disease, peripheral artery disease and rheumatic heart disease. ^3^The global burden of disease define incidence as “the number of new cases of a given cause during a given period in a specified population.”

**Fig B.** **The proportion of deaths from cardiovascular disease, chronic kidney disease and stomach cancer averted each year by food category if all foods were reformulated to UK sodium targets.** Error bars indicate 95% uncertainty intervals. Food categories with greatest potential to prevent total deaths include meat products (n=512 averted deaths), bread (n=140) and cheese (n=96).

**Fig C.** **Effects of full compliance to the Australian government’s sodium targets on averted deaths from cardiovascular disease, chronic kidney disease and stomach cancer estimated by the primary model and in the sensitivity analyses.** Grey bars indicate central estimates of n=1,000 simulations and error bars represent 95% uncertainty intervals around the central estimates. Values inside bars indicate the central estimate as a proportion of the central estimate of the primary analysis. The primary model included a TMREL of 1.6-2.4 g/d and assumption that 85% of daily sodium intakes comes from non-discretionary sources (packaged foods, processed foods and foods consumed out of home).

**References**

1. Australian Bureau of Statistics. 4364.0.55.007 - Australian Health Survey: Nutrition First Results - Foods and Nutrients, 2011-12 Canberra: Australian Bureau of Statistics,; 2014 [cited 2019 17 January]. Available from: <http://www.abs.gov.au/ausstats/abs@.nsf/lookup/4364.0.55.007main+features12011-12>.

2. Australian Government Department of Health. Healthy Food Partnership Reformulation Targets Canberra: Australian Government Department of Health,; 2020 [cited 2020 8 June]. Available from: <https://www1.health.gov.au/internet/main/publishing.nsf/Content/reformulation-targets>.

3. Public Health England. Salt Reduction Targets for 2017 London: Public Health England; 2017 [cited 2020 1 May]. Available from: <https://assets.publishing.service.gov.uk/government/uploads/system/uploads/attachment_data/file/604338/Salt_reduction_targets_for_2017.pdf>.

4. McLean RM. Measuring population sodium intake: a review of methods. nutrients. 2014;6(11):4651-62.

5. Australian Bureau of Statistics. 4363.0.55.001 - Australian Health Survey: Users' Guide, 2011-13. Under-reporting in nutrition surveys Canberra, Australia: Australian Bureau of Statistics,; 2014 [cited 2020 13 May]. Available from: <https://www.abs.gov.au/Ausstats/abs@.nsf/Latestproducts/B42F59546AB32C44CA257CD2001DF4CE?opendocument>.

6. Land MA, Neal B, Johnson C, Nowson C, Margerison C, K.S. P. Salt consumption by Australian adults: a systematic review and meta-analysis. Med J Aust. 2018;208(2):75-81.

7. Nowson C, Lim K, Grimes C, O’Halloran S, Land MA, Webster J, et al. Dietary Salt Intake and Discretionary Salt Use in Two General Population Samples in Australia: 2011 and 2014. Nutrients. 2015;7(12):10501-12. doi: 10.3390/nu7125545.

8. Mattes RD, Donnelly D. Relative contributions of dietary sodium sources. J Am Coll Nutr. 1991;10(4):383-93. doi: 10.1080/07315724.1991.10718167.

9. James WP, Ralph A, Sanchez-Castillo CP. The dominance of salt in manufactured food in the sodium intake of affluent societies. Lancet. 1987;1(8530):426-9.

10. Coyle DH, Shahid M, Dunford EK, Mhurchu CN, McKee S, Santos M, et al. Contribution of major food companies and their products to household dietary sodium purchases in Australia. International Journal of Behavioral Nutrition and Physical Activity. 2020;17(1):81. doi: 10.1186/s12966-020-00982-z.

11. Dunford E, Trevena H, Goodsell C, Ng KH, Webster J, Millis A, et al. FoodSwitch: A Mobile Phone App to Enable Consumers to Make Healthier Food Choices and Crowdsourcing of National Food Composition Data. JMIR Mhealth Uhealth. 2014;2(3):e37. doi: 10.2196/mhealth.3230.

12. The Nielsen Company. Nielsen Consumer Panels 2019 [cited 2019 16 May]. Available from: <https://www.nielsen.com/au/en/solutions/measurement/consumer-panels.html>.

13. Marklund M, Singh G, Greer R, Cudhea F, Matsushita K, Micha R, et al. Estimated population wide benefits and risks in China of lowering sodium through potassium enriched salt substitution: modelling study. 2020;369:m824. doi: 10.1136/bmj.m824 %J BMJ.

14. Singh GM, Danaei G, Farzadfar F, Stevens GA, Woodward M, Wormser D, et al. The Age-Specific Quantitative Effects of Metabolic Risk Factors on Cardiovascular Diseases and Diabetes: A Pooled Analysis. PLOS ONE. 2013;8(7):e65174. doi: 10.1371/journal.pone.0065174.

15. Webb M, Fahimi S, Singh GM, Khatibzadeh S, Micha R, Powles J, et al. Cost effectiveness of a government supported policy strategy to decrease sodium intake: global analysis across 183 nations. BMJ. 2017;356. doi: 10.1136/bmj.i6699.

16. Forouzanfar MH, Liu P, Roth GA, Ng M, Biryukov S, Marczak L, et al. Global Burden of Hypertension and Systolic Blood Pressure of at Least 110 to 115 mm Hg, 1990-2015. JAMA. 2017;317(2):165-82. doi: 10.1001/jama.2016.19043.

17. Webb M, Fahimi S, Singh GM, Khatibzadeh S, Micha R, Powles J, et al. Cost effectiveness of a government supported policy strategy to decrease sodium intake: global analysis across 183 nations. BMJ. 2017;356:i6699. doi: 10.1136/bmj.i6699.

18. Mozaffarian D, Fahimi S, Singh GM, Micha R, Khatibzadeh S, Engell RE, et al. Global sodium consumption and death from cardiovascular causes. N Engl J Med. 2014;371(7):624-34. doi: 10.1056/NEJMoa1304127.

19. D'Elia L, Rossi G, Ippolito R, Cappuccio FP, Strazzullo P. Habitual salt intake and risk of gastric cancer: a meta-analysis of prospective studies. Clin Nutr. 2012;31(4):489-98. doi: 10.1016/j.clnu.2012.01.003.
